# CDK4/6 inhibition initiates cell cycle arrest by nuclear translocation of RB and induces a multistep molecular response

**DOI:** 10.1038/s41420-024-02218-6

**Published:** 2024-10-26

**Authors:** Ting Hong, Anna C. Hogger, Dongbiao Wang, Qi Pan, Julie Gansel, Thomas Engleitner, Rupert Öllinger, Jürgen E. Gschwend, Roland Rad, Roman Nawroth

**Affiliations:** 1 Department of Urology, Klinikum rechts der Isar, Technical University of Munich, Munich, Germany; 2 Present Address: Department of Urology, Shanghai General Hospital, School of Medicine, Shanghai Jiao Tong University, Shanghai, China; 3 Institute of Molecular Oncology and Functional Genomics, Technical University of Munich, Munich, Germany

**Keywords:** Targeted therapies, Transcriptomics, Preclinical research

## Abstract

CDK4/6 inhibitors are standard of care in the treatment of metastatic breast cancer. Treatment regimen consists of a combination with endocrine therapy, since their therapeutic efficacy as monotherapy in most clinical trials was rather limited. Thus, understanding the molecular mechanisms that underlie response to therapy might allow for the development of an improved therapy design. We analyzed the response to the CDK4/6 inhibitor palbociclib in bladder cancer cells over a 48-hour time course using RNA sequencing and identified a multi-step mechanism of response. We next translated these results to the molecular mechanism in bladder cancer cells upon PD treatment. The initial step is characterized by translocation of the RB protein into the nucleus by activation of importin α/β, a mechanism that requires the NLS sequence. In parallel, RB is proteolyzed in the cytoplasm, a process regulated by gankyrin and the SCF complex. Only hypophosphorylated RB accumulates in the nucleus, which is an essential step for an efficient therapy response by initiating G1 arrest. This might explain the poor response in RB negative or mutated patients. At later stages during therapy, increased expression of the MiT/TFE protein family leads to lysosomal biogenesis which is essential to maintain this response. Lastly, cancer cells either undergo senescence and apoptosis or develop mechanisms of resistance following CDK4/6 inhibition.

## Introduction

Inhibition of the cyclin dependent kinases 4 and 6 (CDK4/6) by small molecules in combination with endocrine therapy is standard of care for the treatment of hormone receptor-positive, HER2-negative, advanced or metastatic breast cancer [1]. To date, the three CDK4/6 inhibitors palbociclib (PD), abemaciclib, and ribociclib have been approved by regulatory agencies. However, as monotherapy these compounds are in general not very effective. The reason is that cancer cells can acquire mechanisms of resistance against this therapy. Several molecules and signaling pathways with completely different function have been attributed to contribute to this effect [2, 3]. Thus, it is of interest to identify the hierarchy of molecular mechanisms that induce therapy response.

CDK4/6 inhibitors predominantly induce cell cycle arrest in the G1-phase, followed by a senescence-like phenotype at later stages. Also, alteration in cellular metabolism and enhancement of cancer cell immunogenicity has been reported [3]. Some of these different responses have been observed at distinct time points during treatment, indicating that the response to therapy might be organized along a time kinetics [4–6]. Interestingly, the response or resistance of single cells does not develop homogeneously even in vitro in a cell culture, indicating a certain cellular plasticity in the response to CDK4/6 inhibitors [7, 8].

At the molecular level, CDK4/6 inhibition regulates the activation status of the RB-E2F signaling pathway [9]. Metastatic breast cancer patients who are negative or mutated in RB, do not or only very poorly benefit from treatment with CDK4/6 inhibitors, emphasizing an essential role of RB in this therapy [10]. Depending on its subcellular localization, RB regulates different aspects of cellular biology [11]. In a quiescent state, RB binds to the protein family of E2F transcription factors in a non-phosphorylated form and prevents the induction of cell cycle progression. Following phosphorylation of RB by CDKs, E2Fs are released and facilitate entry into S phase. Besides its role as a regulator of S-phase progression in the nucleus, RB is also localized in the cytoplasm, where it can modulate mitochondrial functions and induce apoptosis [12, 13]. Activation of cell cycle progression is controlled by the ubiquitin-proteasome system (UPS). This also involves the SCF (Skp1-Cul1-F-box protein) E3 ubiquitin ligase complex, which controls cell cycle progression from G1 to early M phase and targets proteins for proteolysis [14]. Activation of SCF is determined by neddylation of Cullin 1 (Cul1), which facilitates interaction with the F-box protein and subsequent ubiquitination of the substrate [15, 16]. It has been extensively documented that CDK4/6 inhibitors not only cause dephosphorylation of RB, but that this response is almost always accompanied by a decrease in RB protein levels [17–19]. This raises questions about the mechanism of action of CDK4/6 inhibitors, since the decrease in RB protein levels should result in the release of E2Fs, thereby initiating the opposite process, meaning the progression of the cell cycle to S-phase.

In this study, we addressed the question of how the response to therapy with CDK4/6 inhibition is organized along a temporal kinetics and which molecular elements are essential components during this response. To this end, we performed a transcriptome analysis in bladder cancer cells treated with the CDK4/6 inhibitor PD at six time points from 0–48h after treatment. Our data allowed us to identify a temporally defined multistep response. When analyzing early time points, we demonstrate proteolysis of cytoplasmically localized RB as well as subcellular translocation of hypophosphorylated RB into the nucleus mediated by importin α/β (KPNA/B) and its accumulation. The increase in nuclear RB levels is responsible for initiation of cell cycle arrest in G1-phase and significantly improves the cellular response to therapy. This finding explains the insufficient response of patients and preclinical models with RB deficiency to CDK4/6 inhibitors. It also enables the development of novel and more effective combination strategies, as we demonstrate using a combination of the nuclear export inhibitor leptomycin B and PD.

## Results

### The molecular response to the CDK4/6 inhibitor palbociclib can be divided into distinct steps

**Fig. 1:**
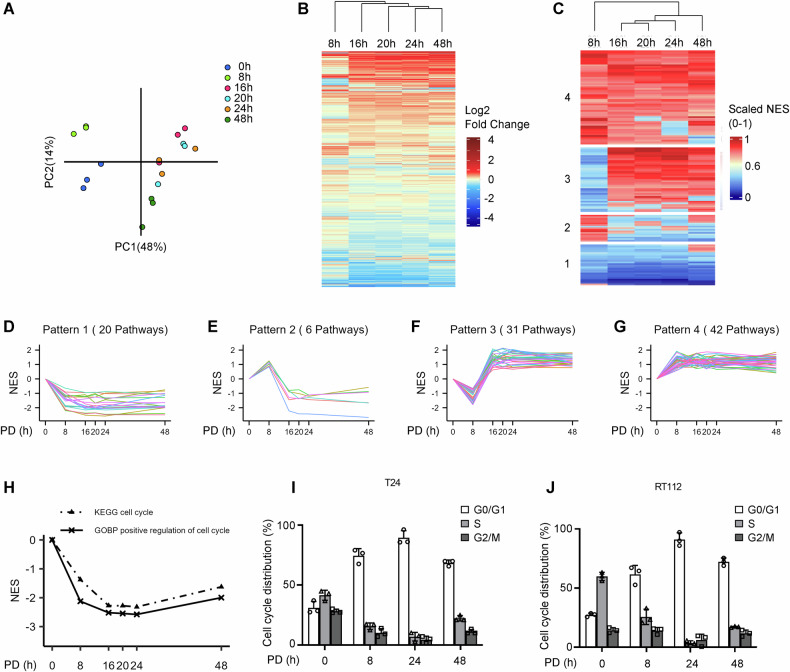
Therapy response to palbociclib treatment can be divided into two distinct steps. T24 cells were treated with 1µM PD and harvested at 8, 16, 20, 24, and 48h for RNA sequencing. **A** PCA shows replicate clustering and separation of time points. PC Principal component. **B** Heatmap displaying the Log2 fold change of gene expression at the indicated timepoints, compared to the untreated 0-hour control. Rows are clusterd based on Euclidean distance. **C** Heatmap displaying the scaled (0–1) normalized enrichment scores of KEGG pathways at the indicated time points compared to the untreated 0-hour control. K-mean clustering revealed four major clusters of differentially expressed pathways. **D**–**G** CDK4/6 inhibition differentially regulates signaling pathways following four major distinct patterns. KEGG pathways were scanned for pathways that fit one of the four major expression patterns observed in the pathway enrichment analysis. **H** Time kinetics based on normalized enrichment score compared with the untreated 0-hour control for cell cycle-related pathways of KEGG and GOBP database. **I**, **J** RT112 and T24 cells were analyzed by flow cytometry at the indicated time points after treatment with 1µM PD. Cell cycle progression was assessed by EdU incorporation and 7-AAD staining. Values are ± SD as a percentage of total cells and represent three independent replicates. NES: normalized enrichment score.

We used RNA sequencing to investigate the temporal sequence of transcriptomic changes in response to PD in T24 bladder cancer cells (Table S1). Principal Component Analysis (PCA) demonstrated that distinct clusters of sample groups formed at the 0, 8 and 48h time points, while the other time points could not be separated from each other, indicating homogeneity of replicates within certain time points (Fig. 1A). This could also indicate that the response to therapy can be divided into early, mid and late time points. For further investigation, a differential gene expression analysis was performed and the expression dynamics of a total of 12 462 differentially expressed genes (DEGs) were identified over the whole time period and visualized based on the Euclidean distance clustering (Fig. 1B). DEGs with absolute log2 fold changes (FC) greater than 1 were considered to be strongly differentially expressed (sDEGs). sDEGs were distributed with 12.5% (1544) at 8h and about 20% (2469–2607 DEGs) at each of the later time points (Table S2). The column dendrogram for differential gene expression analysis shows a difference of the 8-hour time point to the later time points, with the 24 and 48h time points being the most remote ones (Fig. 1B).

This trend was also reflected at the pathway level with a gene set enrichment analysis (GSEA) using the KEGG and GOBP database. The results were summarized in a heatmap based on K-means clustering (Fig. 1C and Fig. S1). 144 signaling pathways in the KEGG and 3117 in the GOBP gene sets were differentially regulated. The column dendrogram revealed a clear separation between the 8-hour time point from the 16–24-hour time range. The 48-hour time point was distinct from all others.

### CDK4/6 inhibition differentially regulates signaling pathways following four patterns

We examined the KEGG-based GSEA, comparing the normalized enrichment scores of each time point with the 0-hour untreated control to generate time kinetic profiles for each pathway. 99 (KEGG) and 2238 (GOBP) pathways could be assigned to four major patterns that corresponded to the respective main clusters identified in GSEA (Fig. S1, Table S3). Pattern 1 comprised 20 pathways that exhibited consistent downregulation, including pathways directly involved in cell cycle progression, DNA replication, and associated repair mechanisms (Fig. 1D).

In pattern 2, we identified 6 pathways defined by initial upregulation at 8h and subsequent downregulation at later time points, including, RNA polymerase and spliceosome, and ubiquitin-mediated proteolysis (Fig. 1E).

31 KEGG pathways followed pattern 3, which is defined by downregulation at 8h followed by upregulation at the later time points, including metabolic pathways and PI3K signaling pathways (Fig. 1F). Pattern 4 displayed consistently upregulated pathways, comprising mostly immunoregulatory processes (Fig. 1G). No specific pattern was evident for 45 signaling pathways (Table S3).

The inhibition of cell cycle progression resulting from CDK4/6 inhibition is well described and one of the main reasons for the observed therapeutic response in RB-positive tumor cells [3, 18]. 942 of 12 462 DEGs are involved in cell cycle regulation according to six relevant KEGG pathways (Cell cycle (Fig. 1H) and p53 signaling pathway in pattern 1, PI3K-Akt signaling pathway in pattern 3, JAK-STAT signaling pathway in pattern 4, MAPK and wnt signaling pathway with no defined pattern assigned).

In the next step, we validated the transcriptomics data at the functional level in the T24 and RT112 cell lines. Cell cycle regulation displayed rapid downregulation but partial recovery after 48h according to the KEGG and the GOBP database GSEA (Fig. 1H) which we confirmed by EdU incorporation assays and 7-AAD DNA staining, analyzed by FACS (Figs. 1I, J, S1F).

In addition, we extended our analysis of pathways associated with pattern 1 to the Reactome and GOBP database and analyzed our cellular senescence dataset as an example of a late response (Fig. S2A). We confirmed this pattern in the T24 and RT112 cell lines by cell body enlargement and senescence-associated beta-galactosidase (SA-βgal) staining, which started 4 days after PD treatment. After 14 days of treatment, a cellular subfraction including 22–45% of T24 and RT112 cells showed positive staining (Fig. S2B). This heterogeneous response was confirmed in a colony formation assay, where some cellular clones persisted and formed colonies that were similar in size compared to control (Fig. S2C).

### Lysosome biogenesis is an essential step in maintaining the response to CDK4/6 inhibition

**Fig. 2:**
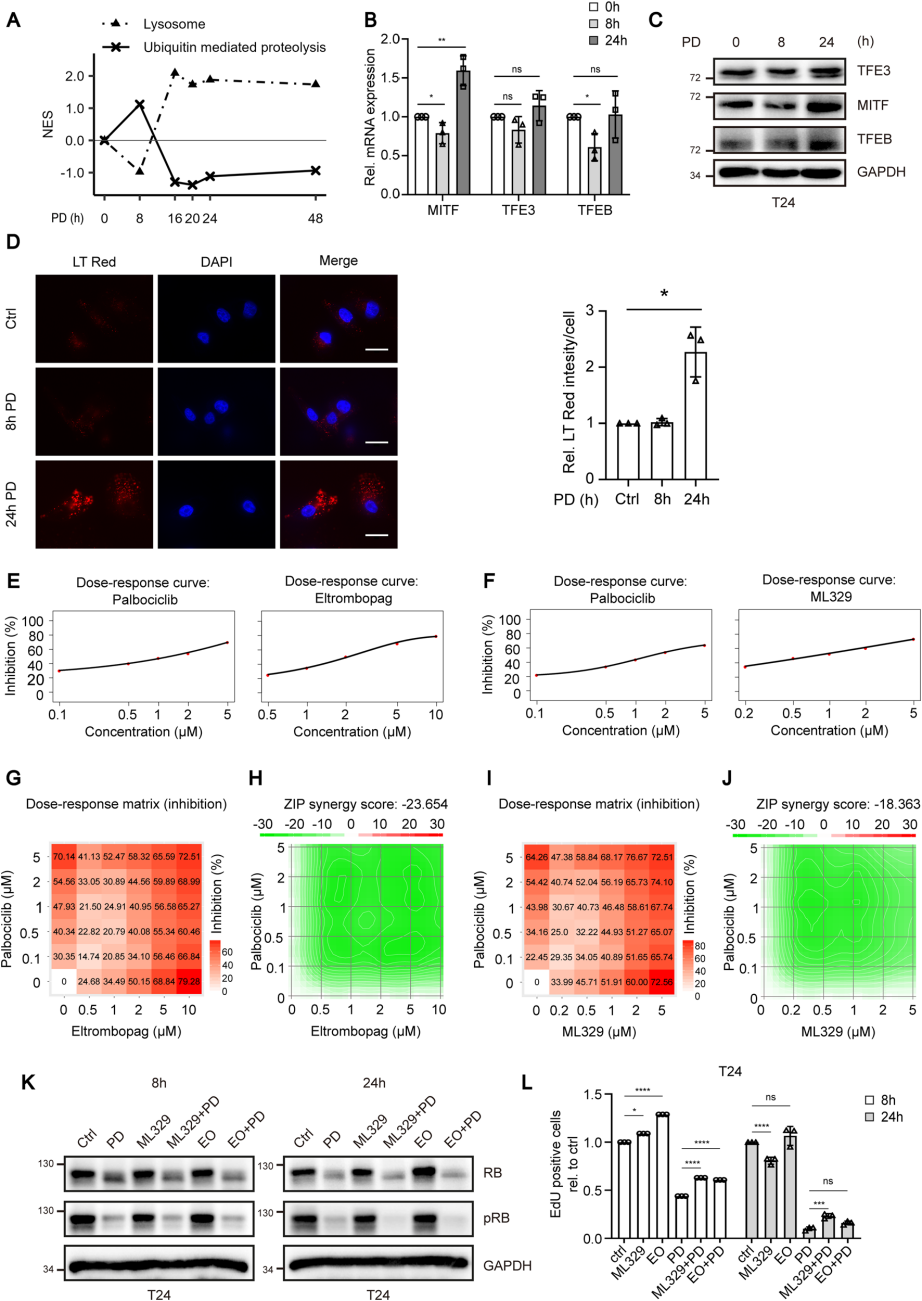
Lysosomal biogenesis supports the maintenance of CDK4/6 inhibitor response. **A** Time kinetics based on normalized enrichment score compared with the untreated 0-h control for KEGG lysosome and KEGG ubiquitin-mediated proteolysis pathways. **B** Relative mRNA expression of MITF, TFE3, and TFEB after 0, 8 and 24h after treatment with 1µM PD. Error bars indicate SD. **C** T24 cells were treated with 1 µM PD for 0, 8, and 24h. Protein expression of transcription factors driving lysosomal biogenesis was analyzed by immunoblotting. **D** Representative microscope images depicting lysosome staining in T24 cells 0, 8, and 24h after treatment with 1µM PD (scale bar equals 20µm) and quantification of LysoTracker Red per cell. > 40 cells were quantified in each group. Error bars indicate SD, asterisk indicates two-tailed Student“s *t* test *p* < 0.05. **E**, **F** Dose–response curves of palbociclib, eltrombopag and ML329 indicating inhibition of cell growth upon different concentrations of inhibitor. **G**, **I** Dose–response matrices and **H**, **J** ZIP synergy score matrices for palbociclib in combination with eltrombopag and ML329. **K** T24 cells were treated with 1µM PD, 5µM ML329, 10µM EO or a combination for 8 and 24h. Protein expression was analyzed by immunoblotting. **L** Cell cycle analysis was conducted by evaluating EdU incorporation and 7-AAD staining for both monotherapy and combination therapy. The relative proportion of cells in the S-phase was quantified by comparison with the control group. Error bars indicate SD, asterisks indicate ANOVA. *P < 0.05; **P < 0.01; ***P < 0.001, ****P < 0.0001.

The control of cell cycle progression is mediated by molecular mechanisms that control protein stability [20]. A very important regulatory mechanism in cell cycle progression is mediated by the proteasome, in which the APC/C and SCF complexes are involved. Another mechanism described is the lysosomal biogenesis, which has recently been linked to cell mitosis [21]. Interestingly, KEGG pathway enrichment analysis revealed that UPS and lysosomal degradation showed completely opposite activation kinetics following pattern 2 and 3, respectively (Fig. 2A). The lysosomal degradation pathway is regulated by the MiT/TFE family members MITF, TFE3, and TFEB [22]. They are involved in the transcriptional regulation of lysosomal biogenesis and can be induced by CDK4/6 inhibitors [22].

We first validated the expression of these molecules at both, the mRNA and protein levels. Only the MITF expression was clearly increased 24h after PD treatment whereas at the protein level all genes showed an increase after 24h (Fig. 2B, C). At the functional level, we could observe PD induced lysosomal biogenesis only from 24h onwards (Fig. 2D) by staining lysosomes with LysoTracker™ Red DND-99, a fluorescent dye for labeling and tracking acidic organelles in living cells, as described by Yin et al. [22]. To investigate the contribution of lysosome biogenesis to PD therapy response, we combined the TFEB inhibitor eltrombopag or the MITF inhibitor ML329 with PD. The effect of the combination was assessed for synergism based on cell growth using the “Zero Interaction Potency Model” (ZIP), a strategy for analyzing and visualizing data on response to combinations of multiple drugs [23]. To quantify the interaction between two drugs, a delta score is calculated as a measure of the overall shift in interaction potency. A positive score thus indicates synergy, while a negative score indicates antagonism. A delta score of 0.1 represents 10% of response above expectation. As monotherapy, PD and ML329 showed an IC50 of 1µM in T24 cells, while eltrombopag displayed an IC50 of 2µM (Fig. 2E, F). For both combinations, ZIP synergy scores of −23.6 and −18.4 revealed a strong antagonistic effect, indicating that lysosomal biogenesis after 24h is an integral part in maintaining the response to CDK4/6 inhibitors (Fig. 2G–J). We also demonstrated that these inhibitors strongly reduce the PD induced lysosomal biogenesis when combined with both drugs (Fig. S3). The combination of PD with these two inhibitors did not affect the degradation kinetics of total RB compared to PD monotherapy (Fig. 2K). Also, the phosphorylation level of RB was not influenced. However, the S-phase population by EdU/7-AAD staining in FACS analysis was increased 1.5–2 fold compared to PD monotherapy (Fig. 2L).

In conclusion, our data suggest that the induction of lysosomal biogenesis by CDK4/6 inhibitor contributes to the maintenance of the response to PD.

### CDK4/6 inhibitor-induced proteolysis of RB is an early event in therapy response

**Fig. 3:**
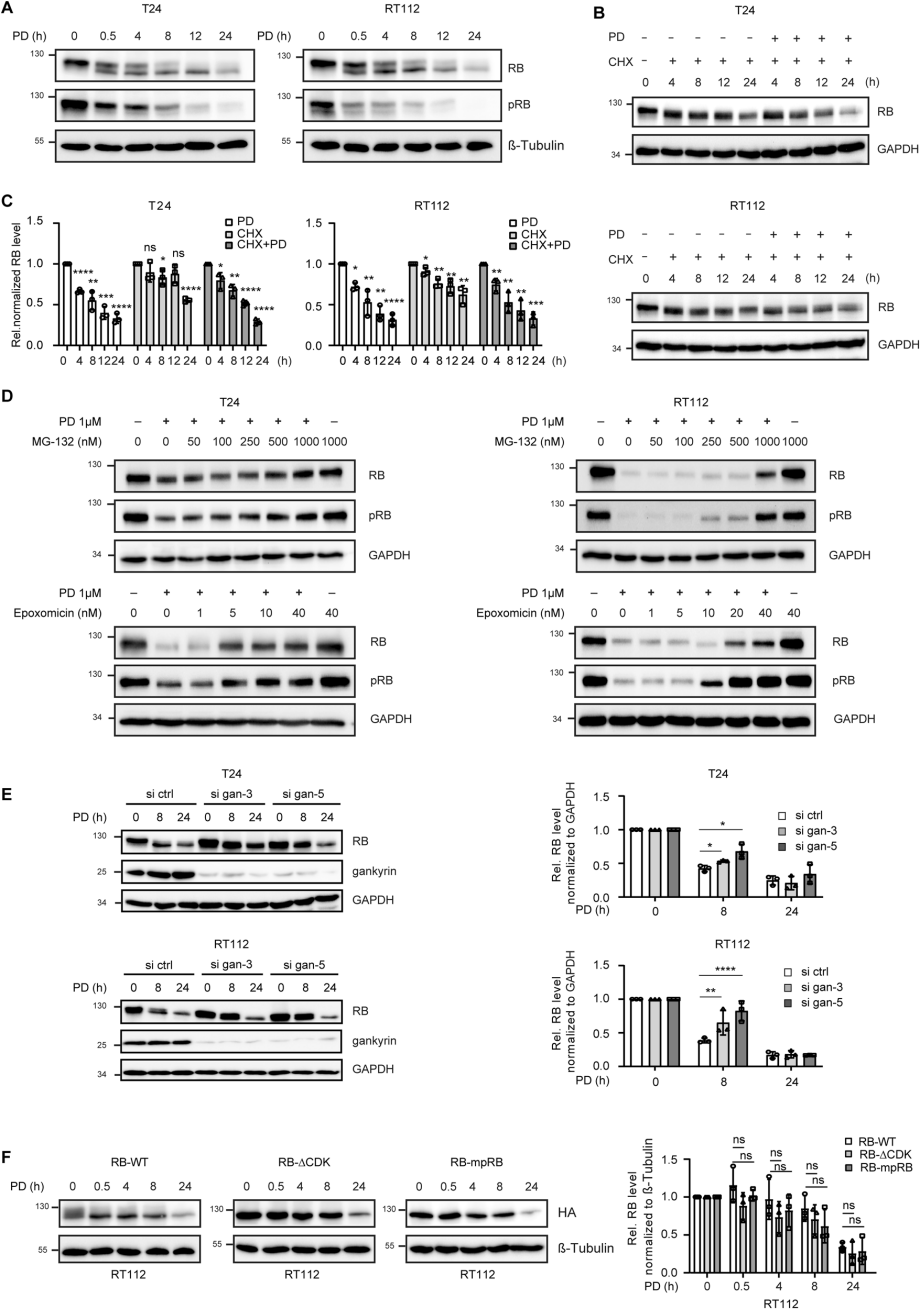
PD induces RB degradation through the proteasomal pathway. **A** T24 and RT112 cells were treated with 1µM PD for 0, 0.5, 4, 8, 12 or 24h. Protein expression was analyzed by immunoblotting. **B** T24 and RT112 cells were treated with 5µg/ml or 20µg/ml CHX alone or in combination with 1µM PD for 0, 4, 8, 12 or 24h. Protein expression was assessed by immunoblotting. A plus sign indicates presence, while a minus sign indicates absence. **C** RB signals in Fig. 3**A** and **B** were quantified densitometrically, normalized to the respective housekeeping protein and displayed relative to 0h. Asterisks indicate a significant difference to the respective 0-hour control. **D** T24 and RT112 cells were treated with 1µM PD for 24h, both with and without MG-132 and epoxomicin as the indicated concentrations. Protein expression was assessed by immunoblotting. **E** T24 and RT112 cells were transfected with control or gankyrin siRNA oligos. After 24h, cells were treated with 1µM PD for 8 or 24h and Western blot analysis was performed using antibodies against RB, gankyrin and GAPDH. RB signals were quantified densitometrically and expressed as the ration si-gankyrin/si-ctrl at each time point after normalized to the respective housekeeping protein. **F** RT112 cells transfected with pCMV HA hRB-wt (RB-WT), pCMV HA hRb delta CDK (RB-ΔCDK) or pCMV HA mpRB (RB-mpRB) plasmids for 24h were then treated with 1µM PD for the indicated hours. Exogenous RB protein was detected by Western blotting. RB signals were quantified densitometrically and expressed as the ratio relative to 0h after normalized to the respective housekeeping protein. Error bars indicate SD, asterisks indicate ANOVA. *P < 0.05; **P < 0.01; ***P < 0.001; ns, not significant.

A central question in this analysis was which molecular mechanisms initiate the response to PD therapy. A key protein in suppressing cell cycle progression is RB. The GSEA data showed an early time-dependent reduction in RB transcript levels, which we validated by mRNA and protein analysis (Fig. S4A, B). At the protein level, we observed a decrease in phosphorylated RB level already 0.5h past treatment (Fig. 3A). Decrease of total RB level started around 4h (Fig. 3A, C). In order to test if this effect was due to degradation or related to the normal half-life of RB, we treated cells with the translational elongation inhibitor CHX with or without PD (Fig. 3B, C). We observed a half-life of RB of about 24h. However, monotherapy with PD shortened the half-life time to only 8–12h. These results suggest that PD actively induces proteolysis of the RB protein at early time points after treatment.

The KEGG pathway analysis (Fig. 2A) also revealed an activation of UPS. For validation of this observation, we first used two proteasome inhibitors, MG-132 or epoxomicin, in combination with PD to inhibit proteasome activity. Tolerable concentrations were determined for these highly cytotoxic compounds in T24 and RT112 cells (Fig. S4C). When combined with PD, a dose-dependent partial rescue of RB level was observed, indicating an involvement of the proteasome in PD-induced RB degradation (Fig. 3D). Since the effective concentrations of the proteasome inhibitors already cause toxicity, we further investigated proteasome-dependent RB proteolysis by knocking down gankyrin, a subunit of the 26S proteasome. Interestingly, we observed a partial rescue of RB, but only within the first 8h of PD treatment (Fig. 3E) [24–26]. The stability of RB has been linked to its phosphorylation level, and it has been published that phosphorylation of RB by CDK4/6 promotes its degradation [27]. We tested this hypothesis by transfecting RT112 cells with a recombinant HA-tagged wild type RB as control (RB-WT), an RB lacking all 15 Ser/Thr CDK acceptor sites (RB-ΔCDK) [28] or a phosphomimetic RB construct in which we modified all CDK acceptor sites (RB-mpRB). Compared to RB-WT, no significant changes in the kinetics of RB proteolysis were observed with RB-ΔCDK and RB-mpRB (Fig. 3F). These data indicate that PD induces RB degradation and this degradation mechanism might be independent of the phosphorylation status of RB.

### SCF ubiquitin ligase complex mediates RB degradation upon PD treatment

**Fig. 4:**
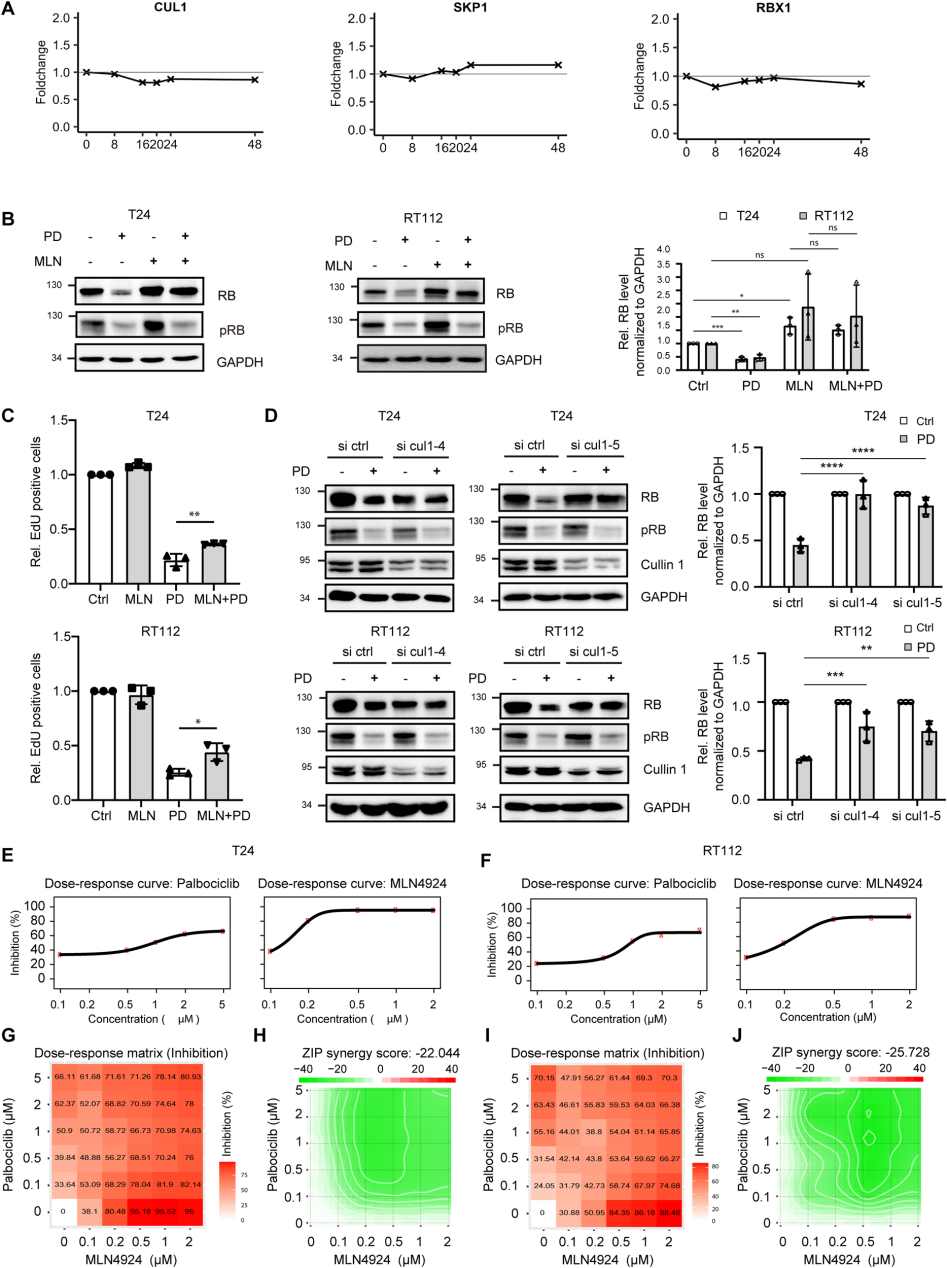
RB gets degraded by SCF ubiquitin Ligase complex upon PD treatment. **A** Differential gene expression analysis of Cul1, SKP1 and RBX1 at indicated time points compared to 0-hour control. **B** Immunoblotting of RB was performed in RT112 and T24 cells treated with 1µM PD for 8h, either in combination with or without 0.2μM MLN4924. RB protein was quantified densitometrically, normalized to GAPDH and the value after PD treatment was compared to the control. **C** T24 and RT112 cells were treated with 1µM PD either in combination with or without 0.2µM MLN4924 for 8h. Cell cycle analysis was assessed by EdU incorporation and 7-AAD staining. **D** T24 and RT112 cells were transfected with control, Cullin 1–4 or Cullin 1–5 siRNA oligos. After 24h, the cells were treated with 1µM PD for 8h, and Western blot analysis was conducted using antibodies against RB, pRB and Cul1. After densitometric analysis, normalized to GAPDH, the values of siRNA were referred to the control. **E**, **F** Dose–response curves were generated for PD or MLN4924 monotherapy in RT112 and T24 cells. **G**, **I** A dose–response matrix was constructed for combination therapy. **H**, **J** An interaction landscape was created based on the dose-response matrix. The ZIP synergy score was calculated for the combination therapy. The dose–response data represent the mean from at least 3 biological replicates. Error bars indicate SD, asterisks indicate ANOVA.*P < 0.05; **P < 0.01; ***P < 0.001, ****P < 0.0001, ns, not significant.

The SCF complex controls the transition from G1 to S-phase, and its activation is regulated by neddylation of Cul1. Accordingly, the NGS data set showed no significant differences of core SCF gene transcripts (Fig. 4A). We used MLN4924 (MLN), a specific small molecule inhibitor of the NEDD8-activating enzyme (NAE) that inactivates Cullin-RING E3 ubiquitin ligases. MLN as monotherapy induced a 1.6-fold increase in RB level, indicating involvement of the SCF complex in the normal turn-over of RB. In combination with PD, after 8h a complete rescue of RB protein levels compared to PD monotherapy was observed in both, RT112 and T24 cells (Fig. 4B, right panel). The PD induced dephosphorylation of RB was not affected by MLN (Fig. 4B). We further conducted cell cycle progression assays and observed strongly antagonistic effects to the PD induced G1 arrest (Fig. 4C). To confirm theses data, we downregulated Cul1 using two specific siRNAs. Again, the combination of Cul1 siRNA and PD resulted in a rescue of RB protein 8h after treatment in both, T24 cells and partially in RT112 cells but RB dephosphorylation was still induced (Fig. 4D). We also analyzed cell viability in the MLN or PD mono- and combination therapy (Fig. 4E, F). In combination therapy, the dose-response matrix showed significantly less suppression of cell viability (Fig. 4G, I) and strong antagonism was calculated in the interaction landscape and the ZIP synergy score (Fig. 4H, J).

Overall, these results indicate that an early event in response to CDK4/6 inhibition is the activation of gankyrin-mediated proteasomal degradation of RB, with a strong contribution of the SCF-E3 complex. The phosphorylation status of RB seems not to be a decisive parameter in this mechanism.

### CDK4/6 inhibition causes nuclear accumulation of RB, which results in G1 arrest

**Fig. 5:**
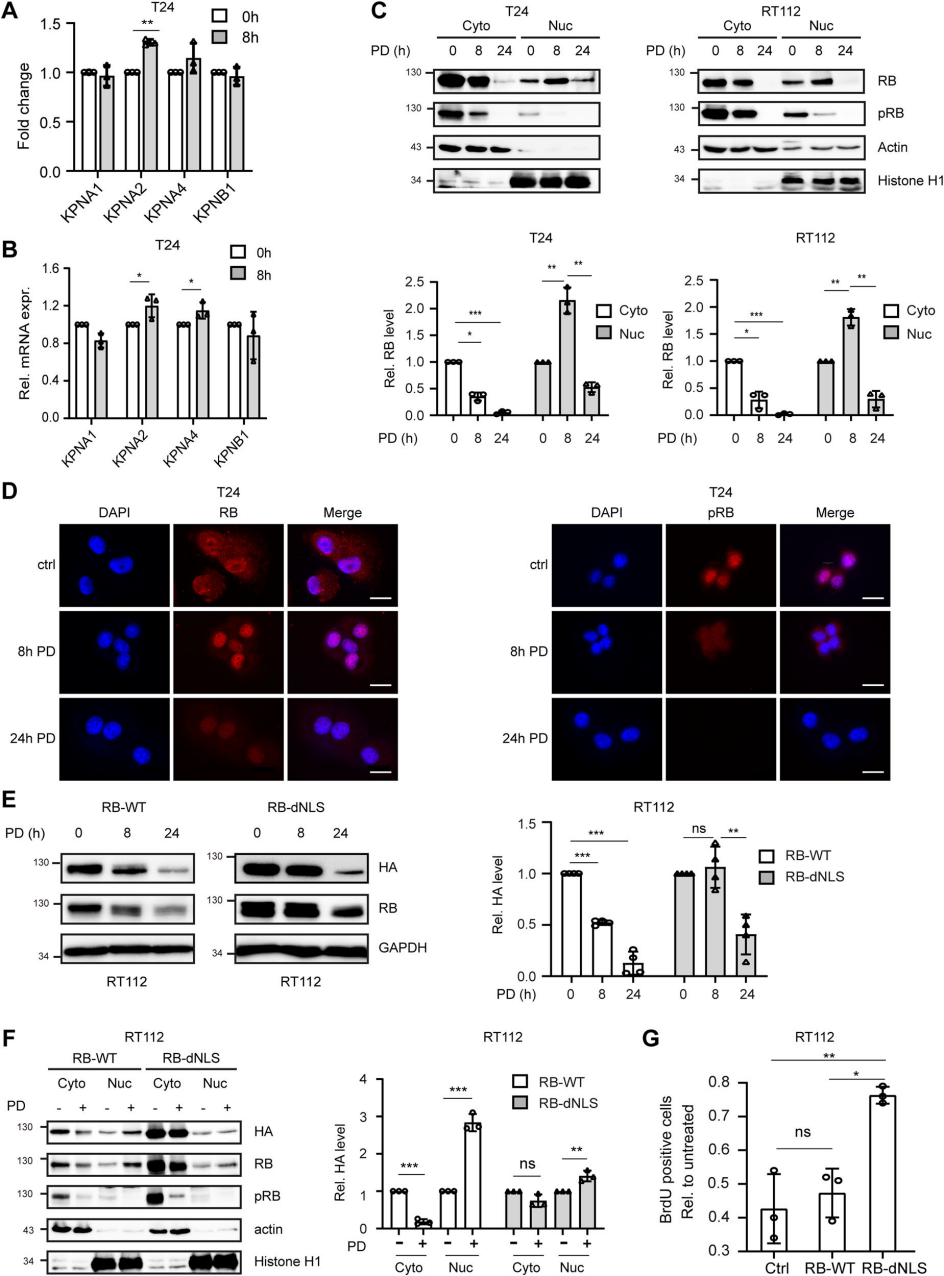
PD induces translocation of RB into the nucleus and inhibits the cell cycle. **A** Differential gene expression analysis was performed for the RNAseq data. The fold change in expression for KPNAs and KPNB1 after 8h compared to the 0-hour control was displayed. **B** T24 cells were treated with 1µM PD for 8h. RT-qPCR was performed to analyze the mRNA expression of these genes. **C** Subcellular localization of RB and pRB proteins in T24 and RT112 cells harvested at indicated time points after 1µM PD treatment was analyzed by Western blot; RB levels are shown as a ratio to the untreated control at 0h after densitometric quantification of Western blot signals. **D** T24 cells were treated with 1µM PD for 0, 8 or 24h. Subcellular localization of RB and pRB was assessed by immunofluorescence staining. Scale bar equals 20µm. **E** RT112 cells transfected with RB-WT or deleted NLS motif (RB-dNLS) plasmids and were treated with 1µM PD for the indicated time points. Expression of endogenous and exogenous RB was determined by Western blotting. Exogenous RB was quantified by densitometry and displayed relative to the untreated 0-hour control. **F** RT112 cells were transfected with RB-WT or RB-dNLS plasmids and then treated with 1µM PD for 8h. Subcellular localization of RB and pRB proteins were analyzed by Western blotting. Exogenous RB was quantified by densitometry and displayed relative to the untreated 0-hour control. **G** RT112 cells were transfected with RB-WT or RB-dNLS plasmids and then treated with 1µM PD for 8h. Cell cycle analysis was assessed by BrdU incorporation and flow cytometry. The analysis of BrdU-positive cells was performed for the ratio after PD treatment compared to the corresponding untreated cells. All quantifications were performed using data (mean±SD) from three independent experiments. Asterisks indicate ANOVA: *P < 0.05; **P < 0.01; ***P < 0.001; ns, not significant.

Although the expression of RB has been appointed to a better response to CDK4/6 inhibitors, the question of why the degradation of RB, and consequently the release of E2Fs, coincides in the G1 arrest and not to progression to S-phase is neglected. One possible explanation might be the subcellular localization of RB during response to therapy. The translocation of RB between the nucleus and the cytoplasm is regulated by the importin α/β1 (Karyopherin A/B1 (KPNA/B1)) complex. RB exhibits in its C-terminus a nuclear localization sequence (NLS) that is recognized by importin α [29]. Importin α comprises a gene family of 7 members (Karyopherin α (KPNA) 1–7) and besides 2 and 7 they all recognize the RB-NLS sequence [30]. Importin β comprises 20 isoforms [31]. In the NGS data, differential gene expression analysis revealed that the genes KPNA2 and 4 were slightly upregulated during early PD treatment (Fig. 5A). To confirm these data, we performed RT-qPCR 8h after PD treatment. The expression of KPNA2 and KPNA4 was increased, while KPNB1 and KPNA1 showed no significant changes (Fig. 5B). Next, we isolated nuclear and cytoplasmic fractions and analyzed the RB protein levels in immunoblots. Upon PD treatment, there was a 60% decrease in RB level in the cytoplasm, while it increased twofold in the nucleus (110% increase in nuclear RB protein), leading to a 50% decrease in total RB level (Fig. 5C, Table S6). Importantly, the nuclear RB was almost completely dephosphorylated. We confirmed these results by immunofluorescence observing RB translocation to the nucleus 8h after PD treatment followed by decreasing RB level at 24h. As a control, we used T24 cells, stably transfected with an shRNA directed against RB (Fig. 5D left panel, Fig. S5) [32]. However, even 24h after treatment the ratio of nuclear to cytoplasmic RB is greatly increased upon PD treatment (Fig. S4D). Phosphorylated RB did not accumulate in the nucleus and gradually diminished throughout treatment (Fig. 5D right panel).

To investigate the rationale behind PD-induced RB translocation, we deleted the NLS motif that is recognized by the KPNA/B1 complex in the RB gene (RB-dNLS) [33]. RB levels in cells transfected with either a recombinant wild-type RB (RB-WT) or RB-dNLS were analyzed. Only the RB-dNLS protein showed stabilization 8h after PD treatment (Fig. 5E). We then analyzed the subcellular distribution of the recombinant RB proteins. After 8h of PD treatment, the wild-type RB exhibited a shift into the nucleus. In contrast, RB-dNLS showed minimal degradation in the cytoplasm and no increase in the nucleus (Fig. 5F). Interestingly, also for the endogenous RB only a very low translocation into the nucleus was observed in the RB-dNLS transfected cells. In cell cycle progression assays, an increase of RB-dNLS expressing cells in S-phase compared with control cells transfected with RB-WT was observed (Fig. 5G). Thus, nuclear translocation and accumulation of RB is an essential step in the initiation of PD-induced G1 arrest.

### Knockdown of proteins of the importin α/β1 (KPNA/B1) complex blocks RB translocation and inhibits the therapeutic effects of PD

**Fig. 6:**
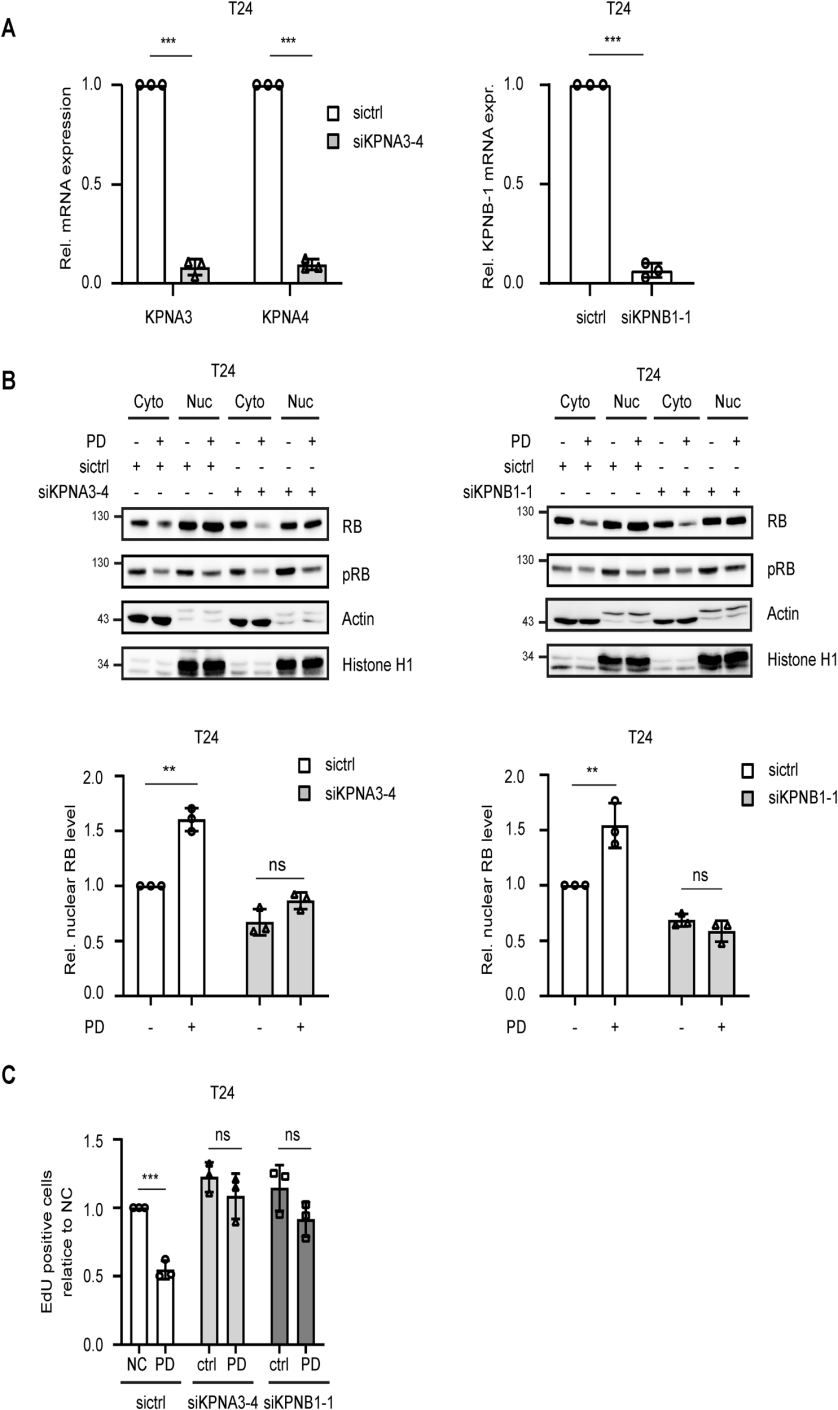
Knockdown of importin complex blocks RB translocation and inhibits the therapeutic effects of PD. **A** mRNA expression of KPNA3, KPNA4 and KPNB1 was assessed 24h past siRNA transfection and displayed relative to control. **B** 24h after transfection with siRNAs, cells were subjected to either PD treatment or not (control). Subcellular localization of RB and pRB was examined by Western blot. The relative amount of RB in the nucleus was referred to the control after densitometric quantification. **C** siRNAs were transfected 24h prior to control or PD treatment. Cell cycle analysis was then performed, and the relative proportion of cells in the S-phase was quantified by comparison with the sictrl (siRNA control) and mock treatment. Error bars indicate SD, asterisks indicate ANOVA. *P < 0.05; **P < 0.01; ***P < 0.001; ns, not significant.

We used siRNAs directed against KPNA (KPNA3/4) and KPNB (KPNB1) which resulted in a knockdown of approximately 90% (Fig. 6A). Both siRNAs inhibited the PD-induced nuclear translocation of RB (Fig. 6B) and antagonized PD induced cell cycle arrest (Fig. 6C). Thus, proteins of the importin α/β1 complex control PD-induced RB translocation from the cytoplasm into the nucleus and this mechanism is essential for the early induction of PD induced cell cycle arrest.

### Inhibition of KPNB1 by IPZ affects the PD-induced RB nuclear translocation and influences the therapeutic efficacy

**Fig. 7:**
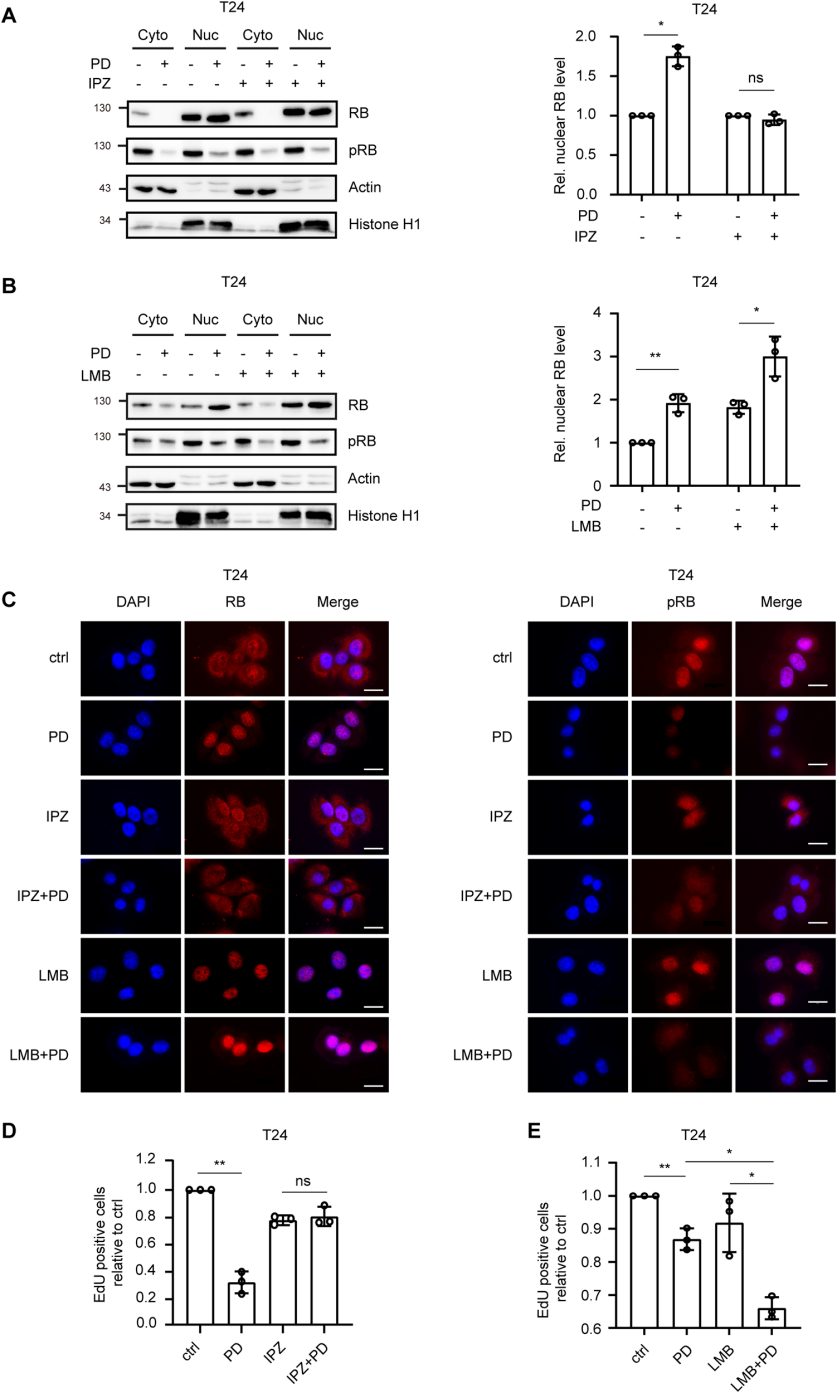
Small molecule inhibitors of nuclear transport proteins interfere with therapy response to PD. **A** T24 cells were treated with 1µM PD with or without 10µM IPZ for 8h. Subcellular localization of RB and pRB was analyzed by Western blotting. RB protein levels in the nucleus are shown as relative values to control after densitometric quantification. **B** T24 cells were treated with 1µM PD with or without 0.5nM LMB. The subcellular localization of RB and pRB was analyzed using Western blotting. RB protein levels in the nucleus are shown as relative values to control after densitometric quantification. **C** T24 cells were treated with PD, IPZ, LMB mono- or combination therapy for 8h. Immunofluorescence staining was used to determine subcellular localization of RB and pRB. Scale bar equals 20µm. **D**, **E** Cell cycle analysis was conducted by evaluating EdU incorporation and 7-AAD for both monotherapy and combination therapy. The relative proportion of cells in the S-phase was quantified by comparison with the control group. All quantifications were performed using data (mean±SD) and analyzed using the ANOVA. Statistical significance was denoted as follows: *P < 0.05; **P < 0.01; ns, not significant.

The nuclear transport protein KPNB can be inhibited by the small molecule importazole (IPZ) [34]. In combination, IPZ completely inhibited the PD-induced RB translocation into the nucleus after 8h of PD treatment (Fig. 7A).

To further test whether the accumulation of RB in the cell nucleus improves response to therapy, the cells were treated with the exportin inhibitor leptomycin B (LMB) alone or in combination with PD. Compared to monotherapy with PD or LMB, the combination led to a 70% increase in nuclear RB levels (Fig. 7B). We then conducted immunofluorescence studies to further examine these results (Fig. 7C). The nuclear translocation of RB induced by PD was inhibited by IPZ. In contrast, LMB treatment resulted in a nuclear accumulation of RB. The Intensity of nuclear RB increased significantly in the combination of LMB and PD. Phosphorylated RB was predominantly detected in the nucleus, independent of cellular treatment (Fig. 7C, right panel).

When examining the consequences on cell cycle progression, the combination with IPZ abolished PD progression of cells in S-phase (Fig. 7D). In contrast, the combination of LMB with PD exerted a significant synergistic impact on inhibiting cell cycle progression into the S-phase (Fig. 7E).

In summary, PD treatment stimulates the translocation and accumulation of dephosphorylated RB in the nucleus. The increase in RB concentration in the nucleus correlates strongly with the blocking of S-phase progression of the cells.

## Discussion

**Fig. 8:**
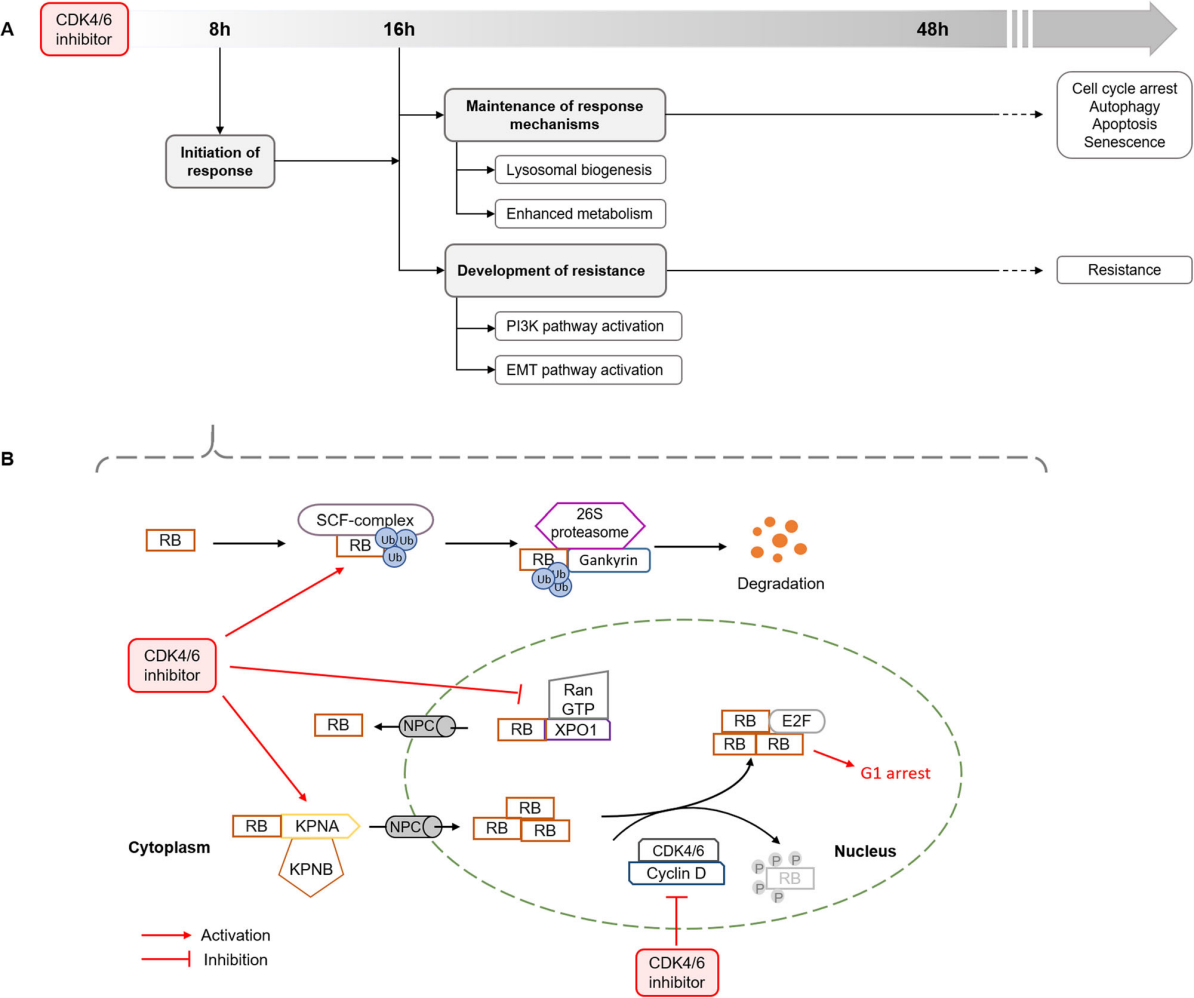
A working model for the molecular response to CDK4/6 inhibition. **A** The therapy response to CDK4/6 inhibitors can be divided into distinct steps: Initiation followed by a plasticity between maintenance of response or development of resistance and finally the separation into a long-term response and resistance. **B** The molecular mechanisms of the initiation. CDK4/6 inhibitor triggers RB proteolysis in the cytosol and nuclear translocation. The accumulation of dephosphorylated RB leads to cell cycle arrest.

The mechanism of response to CDK4/6 inhibitors has been suggested to follow a timely defined, multistep mechanism [4, 35]. In this study, we have first identified these mechanisms in their temporal organization. We also characterized the RB protein-dependent initiation of this response. Using a transcriptomic approach covering 48h of PD treatment, we identified distinct and timely separated steps of the response to therapy that can be divided into an early, RB-dependent response followed by a later second step up to 48h, defined by a plasticity of cells to either maintain the therapy response or develop resistance (Fig. 8). The effects of the CDK4/6 inhibitor PD on the cellular transcriptome have been examined by several research groups, with a predominant focus on the period from 24 to 168h after treatment [36]. In this manuscript, we show that the initiation of therapy response already starts at 4–8h. To analyze the molecular mechanisms underlying the response to CDK4/6 inhibitors, earlier time points must therefore be examined. Within the differentially activated pathways, we identified four major and distinct patterns in their activation status over time. When analyzing the signaling pathways that are differentially regulated during the early step, the most prominent finding was the disruption of the CDK4/6-cyclin D1-RB-E2F axis and cell cycle progression. The strict downregulation (pattern 1) of this pathway continues in the late response. Moreover, the p53 signaling pathway also belongs to this pattern, confirming studies showing that loss of RB and p53 functionality contributes to both intrinsic and acquired resistance [37, 38].

At the molecular level, it is widely acknowledged that the expression of RB in particular is a prerequisite for sensitivity to CDK4/6 inhibitors. RB negative cells do not or only poorly respond to CDK4/6 inhibitors, and even reconstitution of RB negative cell lines with RB does not restore response to therapy [19, 32, 39–41]. CDK4/6 inhibitors reduce protein and transcript levels of RB, which seems to be contradictory to their effect on inducing cell cycle arrest [19]. We demonstrate that the proteasome and lysosome pathway are essential elements in maintaining the response to therapy.

Synergistic effects for simultaneous inhibition of CDK4/6 and lysosome/autophagy functions have been described in the past [21]. Inactivation of CDK4/6 either by small molecules or a genetic knock-down induces lysosomal biogenesis during the cell cycle in S/M-phase by controlling TFEB/TFE3 [22]. Our data show antagonistic effects on PD-induced cell cycle arrest when lysosomal biogenesis is blocked by inhibitors against TFEB and MITF with PD. Lysosomal biogenesis is important for the induction of autophagy, a process that can influence a senescent cell status [42]. This might be an interesting aspect to study in greater detail in order to explain the induction of the senescence phenotype by CDK4/6 inhibitors.

The PD-dependent initiation of G1 arrest depends on the expression of RB. We show here that PD treatment largely affects the stability of RB, a process that is controlled by the activation of the UPS pathway. This mechanism involves gankyrin, a subunit in the 26S proteasome, and the SCF complex. While both pathways have been extensively described for the control of cell cycle progression, their substantial role in initiating the response to CDK4/6 inhibitors is an important finding of this study [43]. However, the F-box protein responsible for the recruitment of RB into the SCF complex has yet to be identified. There is evidence that the phosphorylation level of RB also targets it for proteolysis. Phosphorylation can either provide a protective but also a promoting signal for proteolysis of RB which might be regulated according to specific metabolic conditions [17–19, 27]. This aspect has only partially been addressed for the observed degradation upon CDK4/6 therapy. We did not observe any differences in proteolysis between the RB mutants (lacking or mimicking all described CDK phosphorylation sites in RB) and the wild-type form. Also, when rescuing RB protein level by inactivating the SCF complex, the PD-induced dephosphorylation of RB was not affected. We conclude that the phosphorylation level is not the decisive modification for its PD-induced degradation. Thus, the degradation of RB induced by CDK 4/6 inhibitors might be due to an increase in global proteasome activity [44].

Another alternative to CDK4/6-dependent proteolysis are non-canonical pathways that induce degradation of RB, but these pathways primarily function through modulation of c-Myc and Cip/Kip levels [45].

An emerging aspect of CDK4/6 inhibitors is their effect in modulating an immune response characterized, for instance, by an enhanced capacity for antigen presentation and an increased production and release of cytokines [46–48]. The combination of CDK4/6 inhibitors with immune checkpoint blockade improves the effect of tumor immunotherapy [38, 49]. Our data reveal that this aspect of CDK4/6 inhibitors belongs to the very early events following pattern 4 [50–52].

Following the kinetics of the response to therapy over time until the next step reveals not only the pathways responsible for maintaining the response, but also those that confer resistance. The response mechanisms include the lysosomal pathway, as well as an increase in metabolic pathways that result in the production of reactive oxygen species (ROS). These induce apoptosis and autophagy, providing an explanation for the induction of these processes after 24h of CDK4/6 inhibitor treatment [35, 53, 54]. The PI3K/AKT pathway is also activated in this step and has been described to confer resistance to CDK4/6 inhibitors [55–57]. The combination of CDK4/6 and PI3K inhibitors leads to a synergistic reduction in cell viability and tumor growth and has been reported to overcome drug resistance [55].

Molecular pathways that mediate resistance at this step further include for example the EMT pathway, which is inhibited at 20h of CDK4/6 inhibitor treatment but activated at 48h of treatment [17, 58–60]. In conclusion, there are specific steps in the response to CDK4/6 inhibitors in which cells have the intrinsic plasticity to develop in one direction or another and single cell sequencing would be required for resolving these mechanisms.

In the current model of cell cycle progression, dephosphorylation or degradation of RB results in cell cycle progression into S-phase [61]. Following this model, a decrease in RB level, as induced by CDK4/6 inhibitors, should release E2Fs and induce S-phase progression instead of the observed G1-arrest (Figs. 2 and 3). This is in strong contrast to the current model for regulating the activity of E2Fs and their control in cell cycle progression, and the molecular mechanism that controls E2F activity upon reduced RB levels needs to be clarified.

In this study, we show that an accumulation of hypophosphorylated RB can be observed in the nucleus upon early PD treatment. Most importantly, increasing the level of RB in the nucleus beyond intrinsic RB level inhibits progression into cellular S-phase. The mobilization of RB into the nucleus by PD depends on the NLS binding site within the RB sequence (Fig. 5). In our experiments we observe that in cells transfected with RB-dNLS also the RB level of endogenous RB did not change after PD treatment. It has been described that the pocket domains of RB are involved in nuclear translocation [62], which is probably the reason why PD is less effective in these cells than in the cells transfected with wild-type RB. We speculate that maybe the RB-dNLS can bind to the transporter system but instead of getting transported and released into the nucleus, it occupies and thus blocks this system.

The transport of proteins that display an NLS sequence in the nucleus is mediated by the importin transport receptor complex. We validated involvement of KPNA3, 4 and KPNB1 as protein members of this complex that mediate PD-induced RB translocation into the nucleus (Figs. 6, 7). However, the exact mechanism underlying the increase of this transport has yet to be resolved.

It has to be mentioned that RB-negative cells can respond to CDK4/6 inhibitors in a FOXM1 dependent manner [63]. However, RB negative tumor xenografts respond with significantly less regression compared to RB positive tumors, suggesting a central role of RB in determining the efficacy of the therapy response. Translating these data into clinical observations, there is clear evidence that patients with a negative RB status have no benefit from this therapy [10, 64].

In summary, our results delineate the response mechanism to CDK4/6 inhibitors in distinct steps and demonstrate the role of RB proteolysis and subcellular relocalization in the nucleus as requisites for response to therapy (Fig. 8).

## Materials and methods

### RNA seq

Library preparation for bulk-sequencing of poly(A)-RNA was done as described previously [65]. Briefly, RNA was isolated using Agencourt AMPure XP magnetic beads (Beckman Coulter™, Brea, CA, USA). Barcoded cDNA of each sample was generated with a Maxima RT polymerase (Thermo Fisher Scientific, Waltham, MA, USA) using oligo-dT primer containing barcodes, unique molecular identifiers (UMIs) and an adaptor. 5’-Ends of the cDNAs were extended by a template switch oligo (TSO) and full-length cDNA was amplified with primers binding to the TSO-site and the adaptor. NEBNext^®^ Ultra II FS DNA Library Prep Kit for Illumina (New England Biolabs GmbH, Ipswich, MA, USA) was used to fragment cDNA. After end repair and A-tailing a TruSeq adapter was ligated and 3’-end-fragments were finally amplified using primers with Illumina P5 and P7 overhangs. In comparison to Parekh et al. [65], the P5 and P7 sites were exchanged to allow sequencing of the cDNA in read1 and barcodes and UMIs in read2 to achieve a better cluster recognition. The library was sequenced on a NextSeq 500 (Illumina, San Diego, CA, USA) with 65 cycles for the cDNA in read1 and 19 cycles for the barcodes and UMIs in read2. Data were processed using the published Drop-seq pipeline (v1.0) to generate sample- and gene-wise UMI tables [66]. Reference genome (GRCh38) was used for alignment. Transcript and gene definitions were used according to the GENCODE v38. A differential gene expression analysis was performed and the expression dynamics of a total of 12 462 differentially expressed genes (DEGs) were identified over the 8- to 48-hour period upon CDK4/6 inhibition (Fig. 1C). |log2(fold change) | > 1 was considered as strongly differentially expressed (sDEGs) (Table S2).

### Cell culture

T24 cells were obtained from ATCC (Manassas, VA, USA) and cultured in DMEM high glucose medium containing 10% FBS and 1% penicillin/streptomycin at 37°C in 10% CO_2_. T24 shRB-19 was generated as previously described in our prior work [32]. RT112 cells were obtained from the Leibniz Institute DSMZ (German Collection of Microorganisms and Cell Cultures, Braunschweig, Germany) and maintained in RPMI medium containing 10% FBS, 1% NEEA and 1% penicillin/streptomycin at 37°C in 5% CO_2_. All cells were tested for mycoplasma on a PCR basis by Eurofins Scientific (mycoplasmacheck).

### Treatment of cells

Palbociclib isethionate (PD-0332991, PD) (MedChemExpress LLC (MCE), Princeton, NJ, USA) was dissolved in sterile water as a 10mM stock solution. MG-132, epoxomicin, eltrombopag (SB-497115, EO), ML329, importazole, leptomycin B (LMB) and pevonedistat (MLN4924) were obtained from MCE and cycloheximide (CHX) was obtained from Merck (Merck KGaA, Darmstadt, Germany) dissolved in DMSO and stored as stock solution according to the manufacturer’s recommendation at −20°C and used freshly. The highest DMSO concentration was used as a control for inhibitors dissolved in DMSO. 0.5–2 × 10^4^/ml cells were seeded into cell culture dishes. After treatment, cells were harvested at indicated time points.

### Cell viability assay

Cells were seeded in 24-well plates at 0.5–1.5 × 10^4^ cells per well. Following a 72h treatment, the cells were fixed with 10% cold trichloroacetic acid at 4°C for 1h. After fixation, the cells were washed with H_2_O and stained with 0.05% sulforhodamine B (SRB) (Sigma-Aldrich Chemie GmbH, Munich, Germany) in 1% acetic acid for 30 minutes at room temperature. Subsequently, the cells were washed with 1% acetic acid cells and air-dried. The cell-bound dye was dissolved in 10mM Tris-base solution and the absorbance was measured at 560nm using a Victor X3 microplate reader (Perkin Elmer, Waltham, MA, USA).

### Cell cycle analysis

For cell cycle progression, the Click-it EdU Alexa Fluor 488 flow cytometry kit (Thermo Fisher Scientific, Waltham, MA, USA) and APC BrdU flow kit (BD Biosciences, San Jose, CA, USA) were used according to the manufacturer’s protocol. Briefly, cells were incubated with 10µM Edu or BrdU for 1h and then fixed. After fixation, the cells were washed with 1% BSA in PBS and the EdU staining reaction was performed. For analyzing the DNA content cells were stained with 7-AAD after washing with 1% BSA in PBS and analyzed using the BD Accuri™ C6 Plus Flow Cytometer (BD Biosciences, San Jose, CA, USA) and data processed using FlowJo software (FlowJo LLC, Ashland, OR, USA).

### Colony formation assay

500 cells were seeded in 6-well plates and incubated for 14 days without (control) or with 1µM PD. Colonies were then fixed and stained with crystal violet. Images were captured using an Invitrogen Evos M500 microscope. Images were analyzed with ImageJ (v.1.53t).

### SA-βGAL staining

The assay was performed as previously described [2]. In brief, subconfluent cells were washed gently twice with PBS and then fixed with 2% formaldehyde/ 0.2% glutarahdehyde solution for 5 minutes at room temperature. The fixed cells were washed twice with PBS and then incubated with 500µl of freshly prepared staining solution at 37°C for 12–16h. Plates were sealed with parafilm, protected from light and pH was controlled. Stained cells were washed twice with PBS and once with methanol. 10 random fields in a phase contrast microscope (Axiocam ERc5s, Zeiss) were analyzed using the software Zen Lite 2012 (Carl Zeiss, Oberkochen, Germany).

### Examination of lysosomes

Cells were grown on coverslips and treated with individual compounds as indicated for 8 or 24h. The cells were further cultured in fresh medium containing 0.3µM LysoTracker Red DND-99 (Thermo Fischer Scientific, Waltham, MA, USA) for 30 minutes. Then cells were washed twice with PBS and fixed with 4% paraformaldehyde for 15 minutes at room temperature, washed with PBS and mounted using Prolong Gold Antifade Mountant with DAPI (Thermo Fischer Scientific, Waltham, MA, USA). Images were taken with the Evos M500 microscope from Invitrogen and analyzed with ImageJ software version 1.53t [67].

### Plasmids

The plasmids of human pCMV HA hRb-wt (RB-WT) and pCMV HA hRb delta CDK (RB-ΔCDK) were purchased from addgene [28]. All plasmids were verified by sequencing. For cloning pCMV HA hRb-dLNS (RB-dNLS), approximately 0.2 kb of the human RB C-terminal region were amplified by PCR using the primers in supplement table S4. The amplified DNA fragment were digested with MluI, XhoI and NotI and cloned into the MluI/NotI digested of RB-WT. The sequence for the RB phospho-mimicking mutant plasmid pCMV HA mpRB (RB-mpRB) contains mutations from Serine to Aspartate or Threonine to Glutamate in the 15 CDK sites described for the RB-WT and synthesized by Thermo Fisher. This oligonucleotide was cloned via the EcoRV/NotI sites in the RB-WT plasmid, replacing the RB-WT sequence. All plasmids were verified through sequencing.

### Plasmid and siRNA transfection

Transfection of plasmids was performed with FuGENE® HD (Promega, Madison, WI, USA) reagent according to the manufacturer’s protocol. siKPNA3-4: 5’- GGAGAGGAACCACACUGCUUC-3’, siKPNB1-1: 5′-UAGUCUUCGAUCUCCGCC-3′, Hs_CUL1_4 (sicul1-4, SI00053417, Qiagen, Venlo, Netherlands), Hs_CUL1_5 (sicul1-5, SI02225657, Qiagen), Hs_PSMD10_5 (sigan-5, SI03211635, Qiagen) and sigankyrin-3: 5’-AUUCAUCGAUGAUGUCUUGUG-3’ were transfected using Lipofectamine RNAiMax according to manufacturer’s protocol (Thermo Fisher Scientific, Waltham, MA, USA).

### Quantitative real-time polymerase chain reaction

Total RNA was extracted from cells using the mirVana miRNA Isolation Kit (Thermo Fisher Scientific, Waltham, MA, USA). 2µg RNA was used as a template to synthesize cDNA using the High capacity cDNA reverse transcription kit (Thermo Fisher Scientific, Waltham, MA, USA). qPCR was performed using SYBR Green Master mix (Thermo Fisher Scientific, Waltham, MA, USA) on the CFX96 Real time PCR detection system (Bio-Rad Laboratories, Inc., Hercules, CA, USA). Relative quantification was performed using the ∆∆CT-method. GAPDH was used as the endogenous reference gene. The primer sequences are listed in supplementary table 4.

### Immunofluorescence

The cells were cultured directly on coverslips in 12-well plates at a density of 1×10^4 cells and then treated with the corresponding inhibitors. After treatment, the cells were fixed using ice-cold methanol-acetone (1:1) for 15 minutes at −20°C. Subsequently, the fixed cells were blocked with 3% BSA (in PBS) and incubated with either a primary antibody or IgG for 1h at room temperature, followed by incubation with a secondary anti-rabbit Alexa Fluor 488 or 594-conjugated antibody (Thermo Fisher Scientific, Waltham, MA, USA). The slides were mounted using a mounting medium with DAPI (Abcam) and imaged using an Evos M500 microscope (Thermo Fisher Scientific, Waltham, MA, USA). A negative control was included by incubating cells with IgG.

### Cell lysis

Total cell protein was lysed in ice-cold SDS lysis buffer followed by mechanical shearing. For the isolation of cellular subfractions, cells were lysed in cytosol extraction buffer (buffer A: HEPES 10mM, KCl 15mM, MgCl_2_ 2mM, EDTA 0.1mM and 0.2% IGEPAL CA-630) on ice for 10 min, then harvested mechanically, centrifuged and the supernatant was harvested. As for nuclear extraction, the pellet was washed with buffer A followed by buffer B (HEPES 25 mM, KCl 50mM, EDTA 0.1mM, 10% glycerol + 1% IGEPAL CA-630 + 1% Sodium deoxycholat), buffer C (buffer B - IGEPAL CA-630/Sodium deoxycholat) and lysed in buffer B+0.4M NaCl. All buffers were supplemented with Proteinase Inhibitor Cocktail (Roche, Basel, Switzerland) and adjusted to pH 7.6.

### Immunoblotting

Protein concentrations of the lysates were determined using the BCA^TM^ protein assay kit (Thermo Fisher Scientific, Waltham, MA, USA) following the manufacturer’s protocol. Equal amounts of protein were applied to SDS-PAGE and subsequently transferred to PVDF membranes (GE HealthCare Technologies, Chicago, Illinois, USA). The membranes were then blocked with 5% milk in TBST for 1h. Subsequently, primary antibodies (Table S5) were incubated overnight, followed by incubation with secondary antibodies (Table S5) 1h at room temperature. For antigen detection, an enhanced chemiluminescence (ECL) system (Thermo Fisher Scientific, Waltham, MA, USA) was applied. The chemiluminescent signals were visualized using the ChemiDoc^TM^ MP imaging system (Bio-Rad Laboratories Inc., Hercules, CA, USA) which utilizes a charge-coupled device (CCD) detector with a linear response across a broad dynamic range [68, 69]. During the imaging process it was controlled that the detecting saturation point was not exceeded to allow for quantification. The quantification of Western blots was performed using Image lab software (Bio-Rad Laboratories Inc., Hercules, CA, USA).

### ZIP model

The inhibitory effect of different compounds was evaluated using the “Zero Interaction Potency” Model (ZIP) [23] and the online tool “Synergy Finder” (version 2.0) (http://www.synergyfinder.org/).

### Statistical analysis

For all displayed experiments, 3 biological replicates have been conducted. The program GraphPad Prism 9 for Windows (GraphPad Software, Boston, MA USA, www.graphpad.com) was used for statistical analysis. Two-tailed Student’s *t* test, one-way or two-way ANOVA were applied to analyze for statistical significance. *p* < 0.05 was considered statistically significant and center values are defined as mean. Error bars display the SD.

## Supplementary information

collated supplementary material file

original data

## Data Availability

All the data supporting the findings of this study are available within the paper and its supplementary information files. All other data supporting the findings of this study are available from the corresponding author upon reasonable request.

## Code availability

The code used to produce data can be requested from the corresponding author.
