# Supplementary material for: CDK4/6 inhibition initiates cell cycle arrest by nuclear translocation of RB and induces a multistep molecular response
Source: Cell Death Discov. 2024 Oct 26;10:453. doi: 10.1038/s41420-024-02218-6 (PMC11513128; doi:10.1038/s41420-024-02218-6)
Supplement: Supplementary file 1 — collated supplementary material file [file 41420_2024_2218_MOESM1_ESM.pdf]

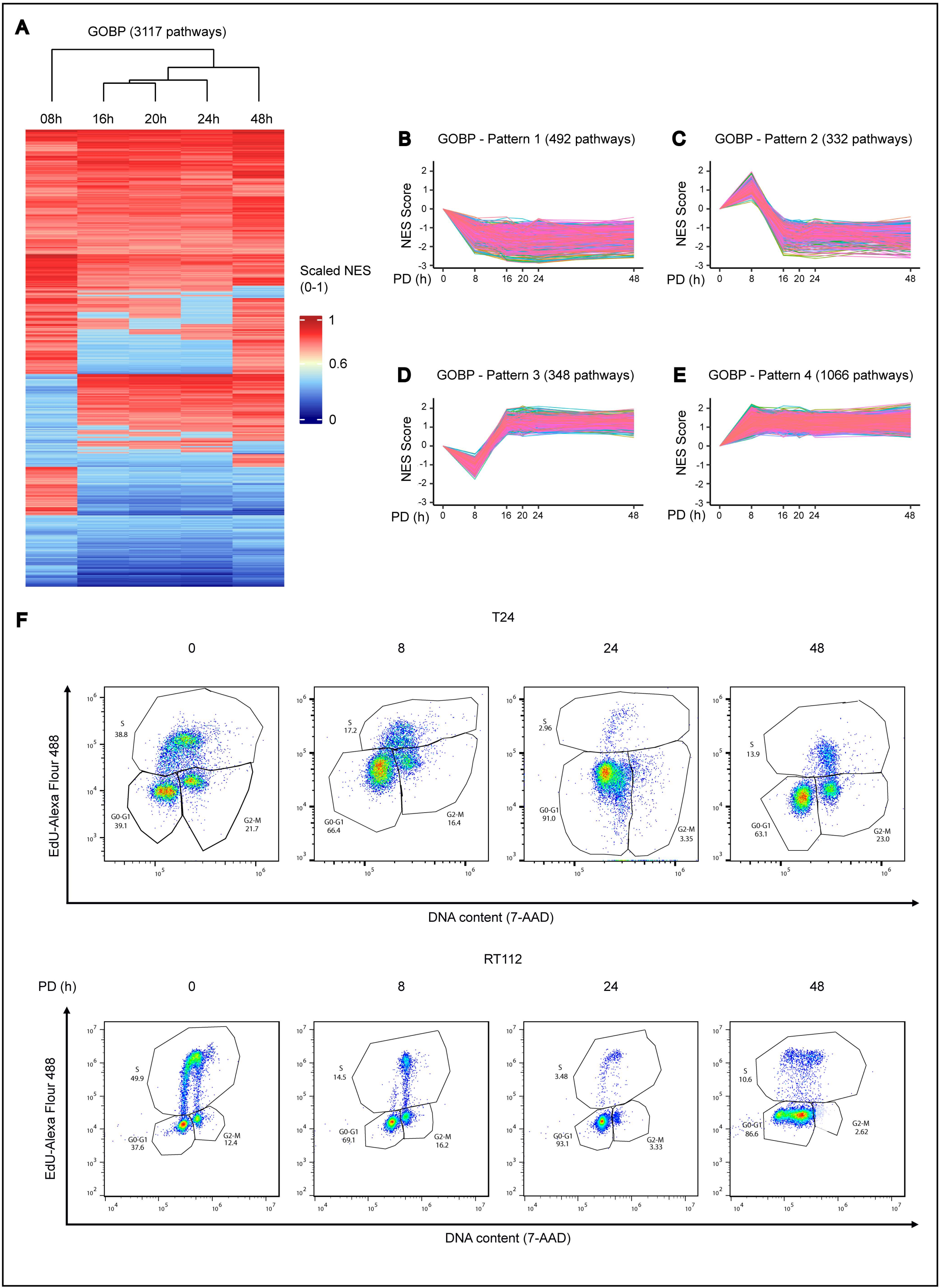
A
GOBP (3117 pathways)
08h
16h
20h
24h
48h
Scaled NES (0-1)
1
0.6
0
B
GOBP - Pattern 1 (492 pathways)
C
GOBP - Pattern 2 (332 pathways)
D
GOBP - Pattern 3 (348 pathways)
E
GOBP - Pattern 4 (1066 pathways)
NES Score
PD (h)
0
8
16
20
24
48
F
T24
0
8
24
48
EdU-Alexa Flour 488
S 38.8
G0-G1 39.1
G2-M 21.7
S 17.2
G0-G1 66.4
G2-M 16.4
S 2.96
G0-G1 91.0
G2-M 3.35
S 13.9
G0-G1 63.1
G2-M 23.0
DNA content (7-AAD)
RT112
PD (h)
0
8
24
48
EdU-Alexa Flour 488
S 49.9
G0-G1 37.6
G2-M 12.4
S 14.5
G0-G1 69.1
G2-M 16.2
S 3.48
G0-G1 93.1
G2-M 3.33
S 10.6
G0-G1 86.6
G2-M 2.62
DNA content (7-AAD)

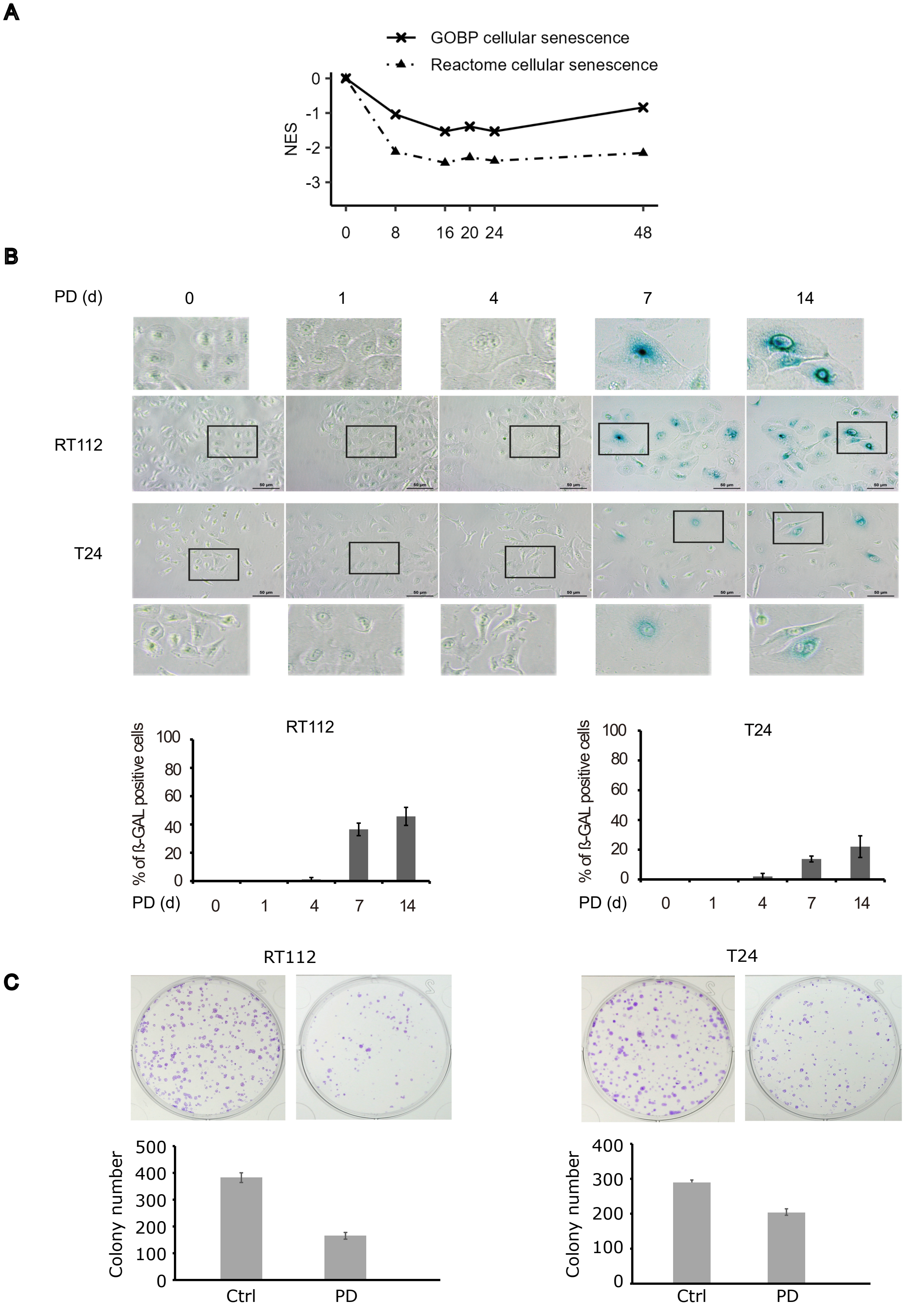
A
GOBP cellular senescence
Reactome cellular senescence
NES
0
-1
-2
-3
0
8
16
20
24
48
B
PD (d)
0
1
4
7
14
RT112
T24
50 μm
RT112
T24
% of ß-GAL positive cells
100
80
60
40
20
0
PD (d)
0
1
4
7
14
C
RT112
T24
Colony number
500
400
300
200
100
0
Ctrl
PD

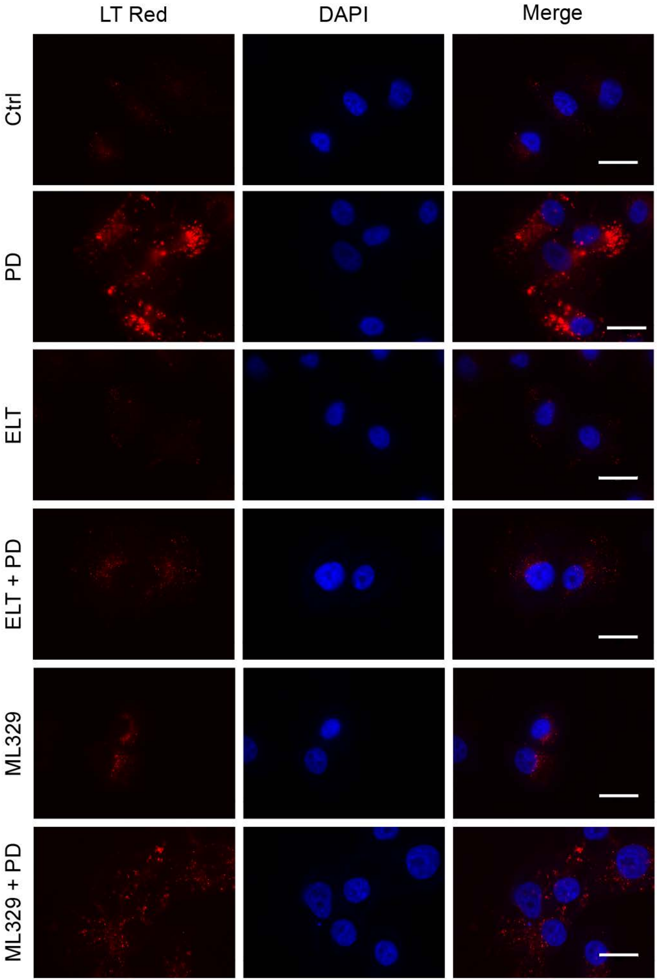
LT Red
DAPI
Merge
Ctrl
PD
ELT
ELT + PD
ML329
ML329 + PD

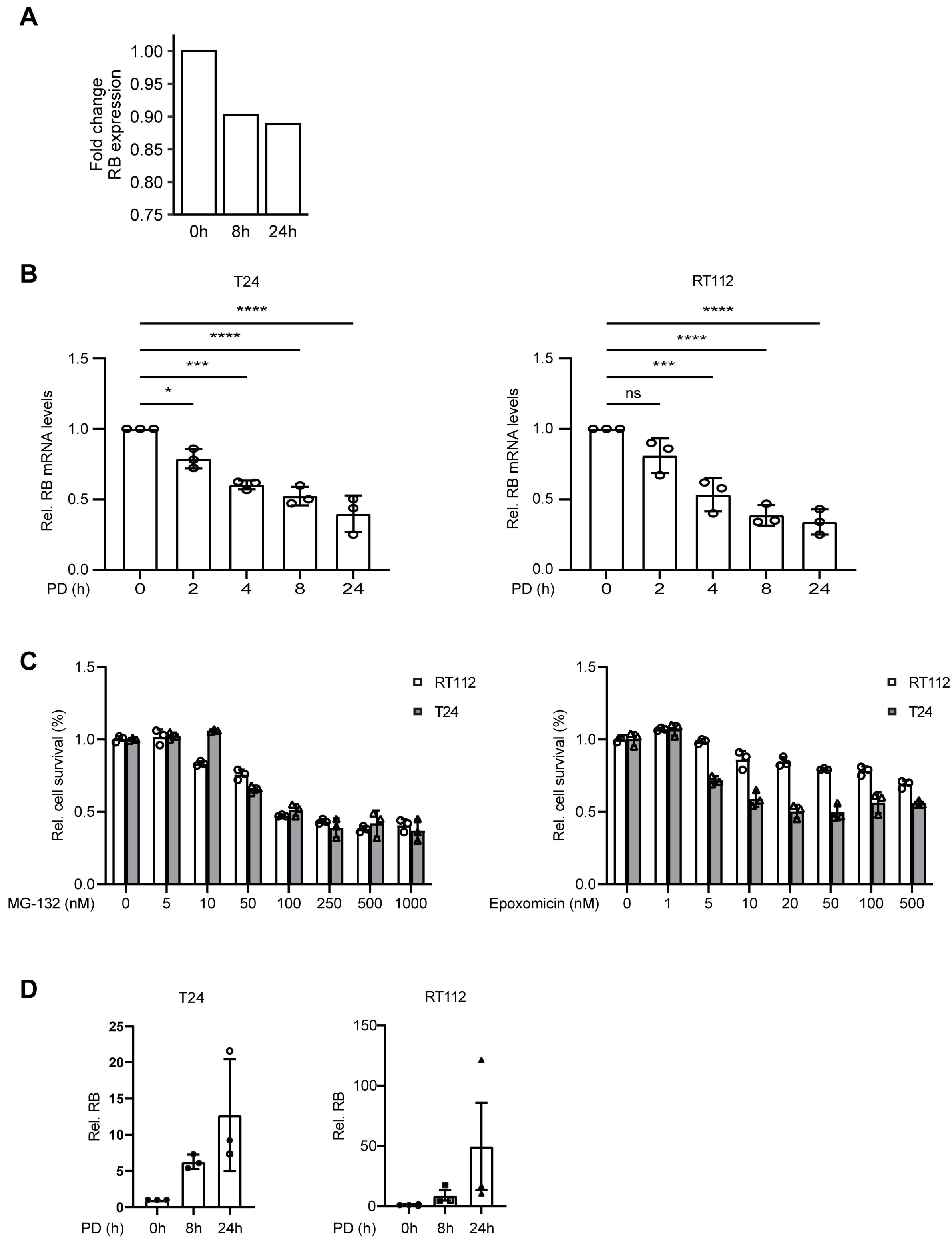

A
Fold change RB expression
1.00
0.95
0.90
0.85
0.80
0.75
0h
8h
24h
B
T24
RT112
Rel. RB mRNA levels
****
***
*
ns
1.5
1.0
0.5
0.0
PD (h)
0
2
4
8
24
C
Rel. cell survival (%)
RT112
T24
MG-132 (nM)
0
5
10
50
100
250
500
1000
Epoxomicin (nM)
1
20
D
Rel. RB
25
20
15
10
150
100
50
0h
8h
24h

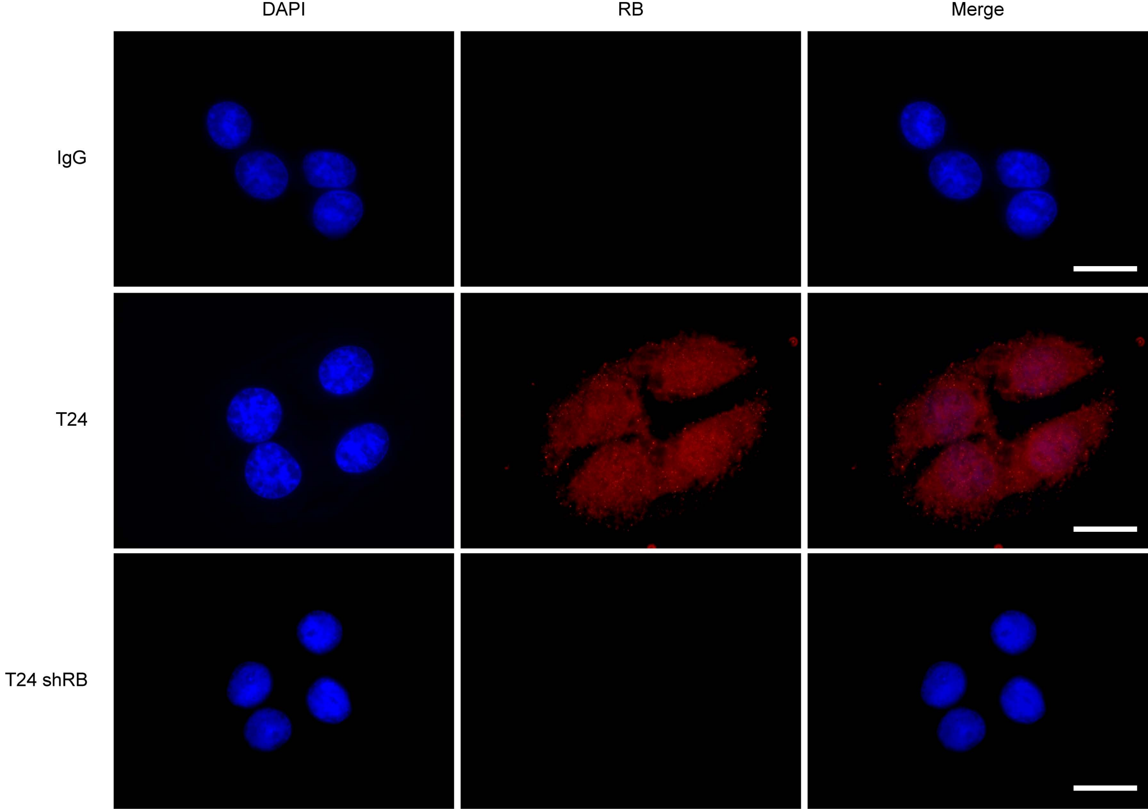
DAPI
RB
Merge
IgG
T24
T24 shRB

NR.	Barcode	Sample	mapped_total	mapped_total_p	unmapped_total	unmapped_total_p
1	AAGATT	c-1	11751258	88.56	1517501	11.44
2	ATTACT	c-2	10636769	88.43	1391042	11.57
3	TAATGA	c-3	9812667	88.27	1303491	11.73
4	TTATTG	pd-8h-1	11367404	88.79	1435488	11.21
5	TCTGCA	pd-8h-2	11216634	88.47	1462488	11.53
6	CCGGAC	pd-8h-3	11080528	88.77	1401471	11.23
7	GACCGC	pd-16h-1	11042334	88.96	1369965	11.04
8	GGCGTC	pd-16h-2	8486380	89.25	1022277	10.75
9	AATACA	pd-16h-3	10432377	88.09	1410159	11.91
10	ATTCTA	pd-20h-1	9587660	88.22	1280851	11.78
11	TACTAT	pd-20h-2	10023790	88.51	1301445	11.49
12	TTGAAA	pd-20h-3	12207058	88.33	1612136	11.67
13	CCAACC	pd-24h-1	13450598	88.61	1729519	11.39
14	CCGTCG	pd-24h-2	7029458	87.94	963611	12.06
15	GAGCGG	pd-24h-3	8674657	88.94	1078285	11.06
16	GGGCAG	pd-48h-1	10466876	88.76	1325064	11.24
17	AATCTT	pd-48h-2	6248174	88.2	835562	11.8
18	ATTTCA	pd-48h-3	7821123	88.11	1055346	11.89

	8h	16h	20h	24h	48h
**sDEG_up**	719	1179	1235	1276	1221
**sDEG_down**	825	1396	1372	1193	1276
**sDEG_total**	1544	2575	2607	2469	2497
**mDEG**	10918	9887	9855	9993	9965
**total**	12462	12462	12462	12462	12462

KEGG pathway	08h	16h	20h	24h	48h	Pattern
Aldosterone regulated sodium reabsorption	-0.912578	-0.95854527	-0.9302389	-1.0177001	-1.0557824	Pattern1
Base excision repair	-1.938143	-1.9329206	-1.851346	-1.9663968	-1.9868017	Pattern1
Bladder cancer	-1.2582014	-1.2586905	-1.2628076	-0.98451084	-0.8405546	Pattern1
Cell cycle	-2.123983	-2.51814	-2.5458477	-2.5787454	-1.997916	Pattern1
Colorectal cancer	-1.1259495	-1.2164954	-1.0139806	-1.406784	-0.75327635	Pattern1
Cysteine and methionine metabolism	-1.2173504	-1.0962833	-1.6790639	-1.4454578	-1.8965695	Pattern1
DNA replication	-2.2020745	-2.3774364	-2.3551614	-2.4596496	-2.395499	Pattern1
Drug metabolism other enzymes	-1.1305441	-1.239933	-1.087048	-1.3198969	-1.287332	Pattern1
Homologous recombination	-1.8520212	-1.9490491	-2.0695074	-1.9734895	-1.8550241	Pattern1
Long term potentiation	-0.6457787	-0.94998926	-0.94693875	-0.8809846	-1.0510714	Pattern1
Mismatch repair	-1.9114988	-2.1531622	-2.1056676	-2.1566632	-1.9874212	Pattern1
Nucleotide excision repair	-1.8787587	-1.9797664	-2.0110202	-2.0670855	-1.9425318	Pattern1
Oocyte meiosis	-1.3406671	-2.0736146	-2.086735	-2.055732	-1.8658384	Pattern1
P53 signaling pathway	-1.4282068	-1.8692819	-1.9921395	-1.8947968	-1.1402628	Pattern1
Progesterone mediated oocyte maturation	-1.1730223	-2.0097773	-1.8916229	-1.9092084	-1.6371641	Pattern1
Prostate cancer	-1.5367067	-1.38989	-1.2613513	-1.3631798	-1.0056459	Pattern1
Purine metabolism	-1.3929456	-1.6234924	-1.683312	-1.6260815	-1.6538587	Pattern1
Pyrimidine metabolism	-1.4044412	-1.9444215	-2.0065775	-2.03687	-1.9256392	Pattern1
RNA degradation	-0.9702864	-1.7340468	-1.8892204	-1.7706721	-2.005581	Pattern1
Systemic lupus erythematosus	-2.1518261	-2.4138596	-2.3719194	-2.4871447	-2.4954572	Pattern1
Basal transcription factors	0.89567816	-1.4473356	-1.3434653	-1.4379712	-1.6591051	Pattern 2
Dorso ventral axis formation	1.257466	-0.89933616	-1.017509	-1.2062632	-0.5849276	Pattern 2
Neurotrophin signaling pathway	1.0160203	-0.79938114	-0.75196433	-1.1206645	-0.870572	Pattern 2
RNA polymerase	1.1951083	-0.8219621	-1.3687987	-1.294095	-1.676681	Pattern 2
Spliceosome	0.83995676	-2.2180934	-2.426227	-2.4321587	-2.672446	Pattern 2
Ubiquitin mediated proteolysis	1.1167787	-1.291506	-1.3812937	-1.1149241	-0.9344571	Pattern 2
Alzheimers disease	-1.4510432	1.3315382	1.5807713	1.9443383	1.0735004	Pattern 3
Arginine and proline metabolism	-0.9238406	1.4531913	1.4841822	1.2162406	1.2690713	Pattern 3
Beta alanine metabolism	-1.2769388	0.7933935	0.8722738	0.80774105	0.77779543	Pattern 3
Biosynthesis of unsaturated fatty acids	-1.384504	1.320168	1.3868698	1.2591876	1.0207589	Pattern 3
Cardiac muscle contraction	-0.9962296	1.4411418	1.6530135	1.4141676	1.3626562	Pattern 3
Drug metabolism cytochrome P450	-1.1141435	1.8711672	2.1047826	1.7757846	1.8101622	Pattern 3
Fatty acid metabolism	-1.3820995	1.5703831	1.469668	1.4966072	1.3648132	Pattern 3
Fc gamma r mediated phagocytosis	-0.6755451	1.1484619	1.1800056	0.8145303	1.0603759	Pattern 3
Fructose and mannose metabolism	-0.8800512	1.76041	1.3241662	1.4445115	1.0181577	Pattern 3
Glycerolipid metabolism	-0.7547175	1.3716164	1.1434586	1.5445347	1.1666443	Pattern 3
Glycolysis gluconeogenesis	-1.7198253	1.6166828	1.3026195	1.355767	1.1811105	Pattern 3
Glycosaminoglycan degradation	-0.89635646	1.2485678	1.4906541	1.4731287	1.4256083	Pattern 3
Histidine metabolism	-0.94667125	1.3284409	1.6632787	1.7369448	1.5318394	Pattern 3
Inositol phosphate metabolism	-0.8879686	1.5907657	1.433291	1.5265698	1.4657279	Pattern 3
Insulin signaling pathway	-0.8854359	1.0829004	0.8712387	0.9951559	0.92764837	Pattern 3
Lysosome	-0.97735	2.0902395	1.7294397	1.8801761	1.7343037	Pattern 3
Metabolism of xenobiotics by cytochrome P450	-1.1201993	1.9543406	2.0826883	1.7900083	1.662515	Pattern 3
Nicotinate and nicotinamide metabolism	-1.037419	1.2078058	1.1692953	1.4702241	1.6678014	Pattern 3
Oxidative phosphorylation	-1.5369631	1.3333442	1.8631477	2.0476677	1.1559033	Pattern 3
Parkinsons disease	-1.5095619	1.1317302	1.657137	1.9870816	1.1065034	Pattern 3
Pentose phosphate pathway	-1.7529769	0.87593746	1.1398574	0.9252118	1.1859791	Pattern 3

Peroxisome	-0.8301676	1.886376	2.1370494	1.9905913	1.5253633	Pattern 3
Phosphatidylinositol signaling system	-0.6595367	1.4979177	1.3154004	1.3984534	1.4232498	Pattern 3
Ppar signaling pathway	-0.82960254	1.72496	1.7932842	1.8131412	1.4078563	Pattern 3
Propanoate metabolism	-1.7942207	0.947969	1.1918526	1.3551818	1.357097	Pattern 3
Pyruvate metabolism	-1.6120973	0.9601201	1.2570727	1.2648152	1.1113216	Pattern 3
Starch and sucrose metabolism	-0.7526902	1.1817822	1.0763887	1.1816239	0.86853606	Pattern 3
Tryptophan metabolism	-1.347971	0.9170383	0.8094336	1.0103375	1.1884282	Pattern 3
Type II diabetes mellitus	-0.9868296	0.8275195	0.7452658	0.7703718	1.0112363	Pattern 3
Valine leucine and isoleucine degradation	-1.3765092	1.190868	1.4968693	1.6168038	1.7466879	Pattern 3
Vascular smooth muscle contraction	-0.6990576	0.62523603	0.73410547	0.66966254	1.024792	Pattern 3
ABC transporters	0.92880344	1.2585919	1.2633001	1.4361309	1.3978889	Pattern 4
Acute myeloid leukemia	0.81061304	0.81447273	1.0614871	0.93433994	1.0388654	Pattern 4
Alanine aspartate and glutamate metabolism	0.79000074	0.9626608	1.5576267	1.4543451	1.5649697	Pattern 4
Amino sugar and nucleotide sugar metabolism	1.3324637	1.7737131	1.5024945	1.6650738	0.90780616	Pattern 4
Antigen processing and presentation	1.5390041	1.1136926	1.1100636	1.0080761	1.3263912	Pattern 4
Arrhythmogenic right ventricular cardiomyopathy	0.94956326	1.2683699	1.0078055	0.9769681	1.8478178	Pattern 4
Axon guidance	1.2803421	1.3700852	1.3570623	1.4498295	1.3169185	Pattern 4
Basal cell carcinoma	0.8872108	0.93335867	0.8503172	0.85798603	0.41519254	Pattern 4
Calcium signaling pathway	1.1707108	1.270378	0.9786328	0.95843303	1.1109308	Pattern 4
Cell adhesion molecules cams	0.953133	1.5590048	1.2684803	1.3572061	1.6856419	Pattern 4
Complement and coagulation cascades	0.919393	1.7962437	1.5163176	1.3917463	1.4595063	Pattern 4
Cytokine cytokine receptor interaction	1.8981869	1.3463019	1.3463002	1.2477801	1.3461688	Pattern 4
Endocytosis	1.5509334	1.3126905	1.1304548	1.0824548	1.346069	Pattern 4
Endometrial cancer	0.8607857	1.1640589	0.9741939	0.81135327	0.766448	Pattern 4
Erbb signaling pathway	1.3564512	0.7090913	0.8339949	0.66369003	0.7586688	Pattern 4
Fc epsilon RI signaling pathway	0.876611	1.1176195	1.1778299	0.81839466	0.5430165	Pattern 4
Focal adhesion	1.4825937	1.4821887	1.3528848	1.1803187	1.6923723	Pattern 4
Galactose metabolism	0.8776211	1.7742536	1.3579148	1.6655301	1.3762397	Pattern 4
Glycerophospholipid metabolism	0.6054122	1.2590445	1.3010229	1.4425278	1.0217028	Pattern 4
Glycosaminoglycan biosynthesis chondroitin sulfa	1.2764546	1.733606	1.5009227	1.4809676	1.0887158	Pattern 4
Glycosaminoglycan biosynthesis heparan sulfate	0.86613023	1.0667564	1.2031916	1.0795062	0.7047866	Pattern 4
Hedgehog signaling pathway	1.2690511	0.8866484	0.88948655	1.2699562	0.44104192	Pattern 4
JAK STAT signaling pathway	1.9228456	1.157883	1.1213037	1.2066127	1.3542484	Pattern 4
Leishmania infection	1.582893	1.1323296	1.1625333	0.80738074	1.529718	Pattern 4
Leukocyte transendothelial migration	1.5953931	1.4944255	1.4071709	1.300315	1.5199367	Pattern 4
Natural killer cell mediated cytotoxicity	1.5806829	1.2803966	0.95529145	1.0805424	1.211662	Pattern 4
Neuroactive ligand receptor interaction	0.76696664	0.9777722	0.952522	0.93329406	1.5177633	Pattern 4
Porphyrin and chlorophyll metabolism	1.0991374	1.5499327	1.721426	1.7554499	1.2973534	Pattern 4
Prion diseases	1.4188229	1.0762197	0.6852751	1.1925238	1.3579832	Pattern 4
Rig I like receptor signaling pathway	1.5660783	1.1459935	0.8295473	1.0685502	0.7756413	Pattern 4
Snare interactions in vesicular transport	1.0474411	1.2017825	1.3126254	1.3846791	1.3182628	Pattern 4
Sphingolipid metabolism	0.579942	1.2356695	1.0847793	1.2732017	1.1258979	Pattern 4
Thyroid cancer	0.9050246	1.0857743	1.081067	1.0869572	0.915374	Pattern 4
Tight junction	1.0563272	1.1608514	1.0648637	1.1701293	1.2447445	Pattern 4
Toll like receptor signaling pathway	1.8331279	1.1693285	1.2265029	0.8615837	1.2039489	Pattern 4
Type I diabetes mellitus	0.9599745	1.1190021	1.3620594	1.4370075	1.7016834	Pattern 4
Tyrosine metabolism	0.68792325	1.7691592	1.8770882	1.8371907	1.4804455	Pattern 4
T cell receptor signaling pathway	1.4761705	0.9895776	0.73473585	0.7883717	0.6194987	Pattern 4
Vasopressin regulated water reabsorption	1.2338642	1.2931708	1.1233249	1.0787233	1.2151116	Pattern 4

VEGF signaling pathway	1.243997	1.3346523	1.3580245	1.2203376	0.76827866	Pattern 4
Vibrio cholerae infection	0.83162034	1.3924968	1.2452223	1.109064	1.0247741	Pattern 4
Viral myocarditis	1.2751524	1.0863914	1.0048208	1.3359641	1.4151069	Pattern 4
Adherens junction	-0.90953106	0.72441334	0.8520259	-0.68354666	1.156317	other
Adipocytokine signaling pathway	1.7614146	0.724349	-0.72989696	-0.73365337	-0.8515401	other
Aminoacyl tRNA biosynthesis	1.2654952	1.1397421	0.89822936	0.8213938	-0.5735182	other
Amyotrophic lateral sclerosis als	1.1824867	0.8226673	0.9024589	0.80327696	-1.0140908	other
Apoptosis	1.8597252	0.8785932	-0.61021733	0.90058273	0.8745174	other
Butanoate metabolism	-1.4455812	-0.757278	1.1180742	1.0118433	1.458774	other
B cell receptor signaling pathway	1.4778333	0.9950039	0.9311922	-0.8600382	0.68731755	other
Chemokine signaling pathway	2.0026288	0.8935792	0.7727324	-0.7497255	0.9043129	other
Chronic myeloid leukemia	-1.2459706	-0.91216254	-0.9679517	-1.0063773	1.0338566	other
Citrate cycle tca cycle	-0.98297656	1.1535991	0.81685174	1.1413622	-0.8992798	other
Cytosolic DNA sensing pathway	1.9618268	0.90673065	-0.77420163	0.7170431	1.0270118	other
Dilated cardiomyopathy	0.890115	1.2167405	0.95964795	-0.75301766	1.7416675	other
Ecm receptor interaction	1.0545235	1.1492667	-0.9012556	1.0917871	1.9740654	other
Epithelial cell signaling in helicobacter pylori infec	1.9115456	0.68344635	0.5729173	-1.0365589	0.87568235	other
Gap junction	1.2028788	-0.92798114	0.8023488	-1.1227456	0.86280566	other
Glioma	-1.4766514	-0.91859776	-0.88216805	-0.6656222	0.92767763	other
Glutathione metabolism	-1.3084184	-0.99001646	0.98363835	-0.9041331	1.0922807	other
Glycosylphosphatidylinositol gpi anchor biosynthe	-0.8628175	-0.5046566	-0.7258364	0.8867691	-1.2694381	other
Gnrh signaling pathway	1.3872932	0.7946745	1.0214527	-0.75359684	0.60937154	other
Hematopoietic cell lineage	1.2764696	-0.8916195	-1.1928618	-0.7872549	1.0061811	other
Huntingtons disease	-1.2660891	0.8893252	1.1395588	1.4590822	-0.8164742	other
Hypertrophic cardiomyopathy hcm	1.20778	1.1606278	0.8717266	-0.6169866	1.7504766	other
Long term depression	0.83920115	0.5594725	-0.56818795	0.54701775	-0.673589	other
Lysine degradation	-1.1619095	-1.0887066	-1.1653802	-1.215858	0.57565486	other
Mapk signaling pathway	1.5402154	1.0497627	0.9149604	-0.9584008	0.9186591	other
Melanogenesis	1.2812495	0.609439	0.6088152	0.6345167	-0.67917717	other
Melanoma	-1.4020677	-1.1926917	-1.0695578	-0.9014485	0.6865325	other
Mtor signaling pathway	-0.9037222	0.53346807	-0.60693103	0.72849816	0.5994998	other
Nod like receptor signaling pathway	2.1196892	0.7439629	0.7384803	0.66577166	-0.79505247	other
Non small cell lung cancer	-1.3733075	0.84183514	-0.74147487	0.84101313	0.9033308	other
Notch signaling pathway	1.4466823	0.9529764	-0.7696348	-0.8976781	1.1526772	other
N-glycan biosynthesis	1.4860231	1.1189358	0.93059206	0.91689515	-1.50084	other
Pancreatic cancer	-1.3551474	-0.5920955	0.7985366	-0.7394572	1.4061397	other
Pathogenic escherichia coli infection	1.4251033	0.9812177	1.0631098	-0.91468436	0.7734506	other
Pathways in cancer	1.2062851	-1.0057285	-1.0462036	-1.1176978	1.0403367	other
Proteasome	0.8809385	0.5525172	-0.8694636	-0.62157065	-1.4758755	other
Protein export	1.4320203	-0.6890116	-1.0857208	0.6373689	-1.2623729	other
Regulation of actin cytoskeleton	1.101039	0.8563809	1.1042774	-0.77785224	1.4656088	other
Regulation of autophagy	-0.6831388	-0.6900316	-0.75053	0.97519976	1.0065334	other
Renal cell carcinoma	0.9803317	-0.64656436	0.7507211	-0.8784372	-0.8523289	other
Ribosome	-1.2265586	0.86180353	1.8219649	1.0293233	-1.047165	other
Selenoamino acid metabolism	1.1429284	1.0269276	0.7085889	1.2663817	-0.856273	other
Small cell lung cancer	-1.1949049	-1.0413587	-1.309377	-1.1281933	1.0244107	other
TGF beta signaling pathway	0.92450684	-0.8259393	-0.81342417	-0.5978337	1.0474721	other
Wnt signaling pathway	1.386183	-0.6350623	0.68464285	-0.9940931	-0.6342028	other

Table S4. Primers used in the study

Gene	Forward primer	Reverse primer
Actin	CATGTACGTTGCTATCCAGGC	CTCCTTAATGTCACGCACGAT
GAPDH	TGG CAT GGA CTG TGG TCA TGA G	ACT GGC GTC TTC ACC ACC ATG G
KPNB1	ATC AGG AGA ACG TAC ACC CG	CCC CTA TTA GTC CAG CAG CA
KPNA1	TTC AAA GCA AAA CCG CCT GA	CAG CAA CCA GGA AAG CAC AT
KPNA2	AAG GCT GTG GTA GAT GGA GG	ACC AAG TCT CGG AAC ACT GA
KPNA3	CCT TTC TTG TGC CCC TTC TG	TTG GGA AGT GTG ACA GGA CA
KPNA4	ATT TGG TTC CTC TGC TCA GC	GCT GGG AAG TGT GAA AGA GC
MITF	CGA CAG AAG AAA CTG GAG CAC	AAA TCT GGA GAG CAG AGA CCC
RB	AGC AAC CCT CCT AAA CCA CT	TGT TTG AGG TAT CCA TGC TAT CA
RB-dNLS-1	CAA CTA CGC GTG TAA ATT CTA CTG C	CGC GGA TCC AAT GTG AGG TAT TGG TGA CAA GG
RB-dNLS-2	CGC GGA TCC AAC CCT CCT AAA CCA CTG	CGC GCG GCC GCC ATC ACC TCA TTT CTC TTC CTT
TFE3	GAG ATC TCT GAG ACC GAG GC	GAG AGT GCC CAG TTC CTT GA
TFEB	GGA GAT GAC CAA CAA GCA GC	CCA GCT CAG CCA TGT TCA TG

Table S5. Antibodies used in the study.

Antibody	Catalog number	Company	Dilution
Actin	A2066	Sigma-Aldrich	1/1000
ß-Tubulin	2128	Cell Signaling Technology	1/1000
Cullin 1	32-2400	ThermoFisher Scientific	1/1000
GAPDH	2118	Cell Signaling Technology	1/1000
gankyrin	HPA002920	Sigma-Aldrich	1/1000
HA	2367	Cell Signaling Technology	1/1000
Histone H1	05-457	Millipore	1/1000
MITF	12590	Cell Signaling Technology	1/1000
RB	554136	Cell Signaling Technology	1/500 -1000
pRB (S780)	8180	Cell Signaling Technology	1/1000
pRB (Ser 780)	701272	Invitrogen	1/1000
TFE3	SAB4502953	Sigma-Aldrich	1/1000
TFEB	83010	Cell Signaling Technology	1/1000
IgG mouse	5415	Cell Signaling Technology	1/1000
IgG rabbit	3900	Cell Signaling Technology	1/1000
IgG goat anti-Rabbit Alexa Fluor 488	A11008	ThermoFisher Scientific	1/500
IgG goat anti-Rabbit Alexa Fluor 594	A11037	ThermoFisher Scientific	1/500
IgG goat anti-Mouse Alexa Fluor 594	A11005	ThermoFisher Scientific	1/500
Peroxidaseconjugated anti-mouse IgG	715-036-150	Dianova	1/10000
Peroxidaseconjugated anti-rabbit IgG	711-036-152	Dianova	1/10000

Table S6 Quantification of total and subcellular RB

	Whole-cell lysate		Subcellular structures			
			C	N	C	N
Time (h) / Cell line	0	8	0	0	8	8
T24	1	0.54	0.90	0.10	0.31	0.21
RT112	1	0.55	0.87	0.13	0.32	0.22

C: cytoplasm
N: nucleus

Fig. S1 CDK4/6 inhibition differentially regulates signaling pathways **(A)** Pathway enrichment analysis based on GOBP database. **(B-E)** GOBP pathways were scanned for pathways that follow one of the four primarily observed time kinetic patterns. **(F)** RT112 and T24 cells were analyzed by flow cytometry at the indicated time points after treatment with 1 µM PD. Exemplary gating strategies are displayed for both cell lines.

Fig. S2 Induction of senescence after long-term PD treatment. **(A)** Time kinetics based on normalized enrichment score compared to the untreated 0-hour control for cell cycle related pathways in Reactome and GOBP database. **(B)** Senescent cells were detected by SA-β-Galactosidase staining. Representative images from three independent experiments are displayed. The scale bar equals 50 µm. The percentage of β-Galactosidase positive cells was calculated and displayed +/- SD. Asterisk indicates two-tailed Student t-test $p < 0.05$. **(C)** Colony formation analysis upon PD treatment. T24 and RT112 cells were treated with 1 µM PD or control for 14 days. These cells were analyzed by plate colony formation assay.

Fig. S3 Lysosomal biogenesis supports the maintenance of CDK4/6 inhibitor response. Representative microscope

images depicting lysosome staining in T24 cells treated with 10 μM eltrombopag or 5 μM ML329 and in combination with 1 μM PD for 24 hours. Scale bars represent 20 μm.

Fig S4. PD induces RB degradation through the proteasomal pathway. **(A)** RB gene expression analysis was performed at 8, 24 hours compared to the 0-hour control. **(B)** T24 and RT112 cells were treated with 1 μM PD for indicated hours. The RB mRNA levels were detected by RT-qPCR. The graphs show the values +/- SD from three different experiments. Asterisk indicates two-tailed Student t-test $p < 0.05$. **(C)** T24 and RT112 cells were treated with either MG-132 or epoxomicin of indicated concentrations. Cell viability was detected after 24 hours of the treatment. The graphs show the values +/- S.E. from three different experiments. **(D)** T24 and RT112 cells were treated with PD for 0, 8 or 24 hours. Densitometrically quantification of immunoblots was displayed as the nuclear to cytosol ratio.

Fig S5. The control of immunofluorescence. Subcellular localization of RB was assessed by immunofluorescence staining. The scale bar equals 20 μm.

Table S1 Overview of reads generated by single end RNA sequencing.

Table S2 Summary of the Differential Gene Expression Analysis: Significant upregulated (sDEG_up): $P < 0.05$; $\log2(FC) > 1$; significant downregulated (sDEG_down): $P < 0.05$; $\log2(FC) < -1$; minor differentially expressed (mDEG): $P < 0.05$; $|\log2(FC)| < 1$

Table S3 KEGG pathways were assigned to four different time kinetic pattern based on the normalized enrichment score. Remaining pathways are listed with the label “other”.

Table S4 Primers used in this study

Table S5 Antibodies used in this study

Table S6 Quantification of total and subcellular RB by immunoblots
