# Supplementary material for: CDK4/6 inhibition initiates cell cycle arrest by nuclear translocation of RB and induces a multistep molecular response
Source: Cell Death Discov. 2024 Oct 26;10:453. doi: 10.1038/s41420-024-02218-6 (PMC11513128; doi:10.1038/s41420-024-02218-6)
Supplement: Supplementary file 2 — original data [file 41420_2024_2218_MOESM2_ESM.pdf]

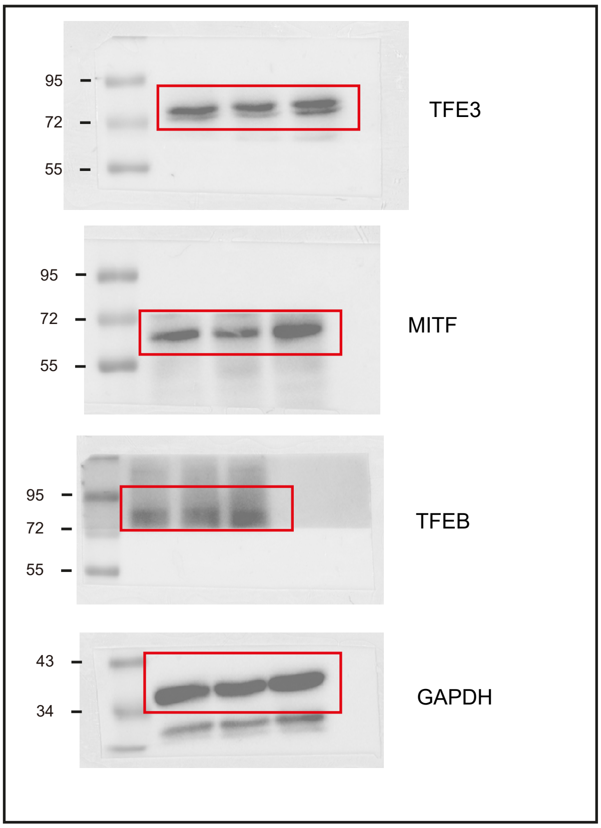
95
72
55
TFE3
95
72
55
MITF
95
72
55
TFEB
43
34
GAPDH

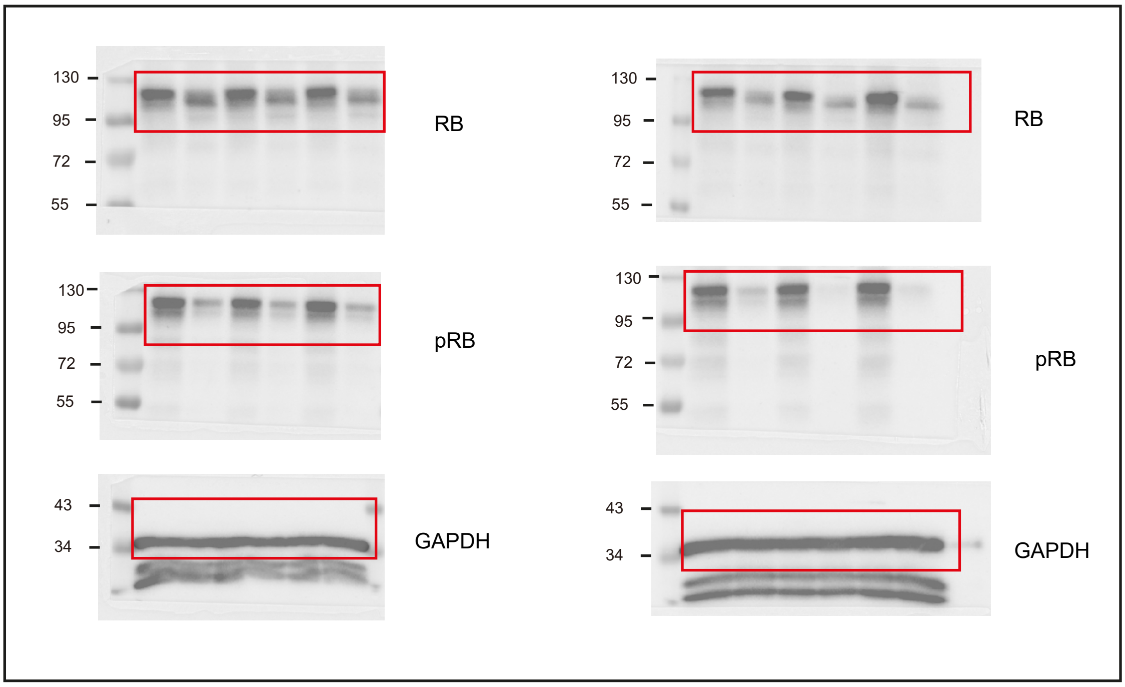
130
95
72
55
RB
130
95
72
55
RB
130
95
72
55
pRB
130
95
72
55
pRB
43
34
GAPDH
43
34
GAPDH

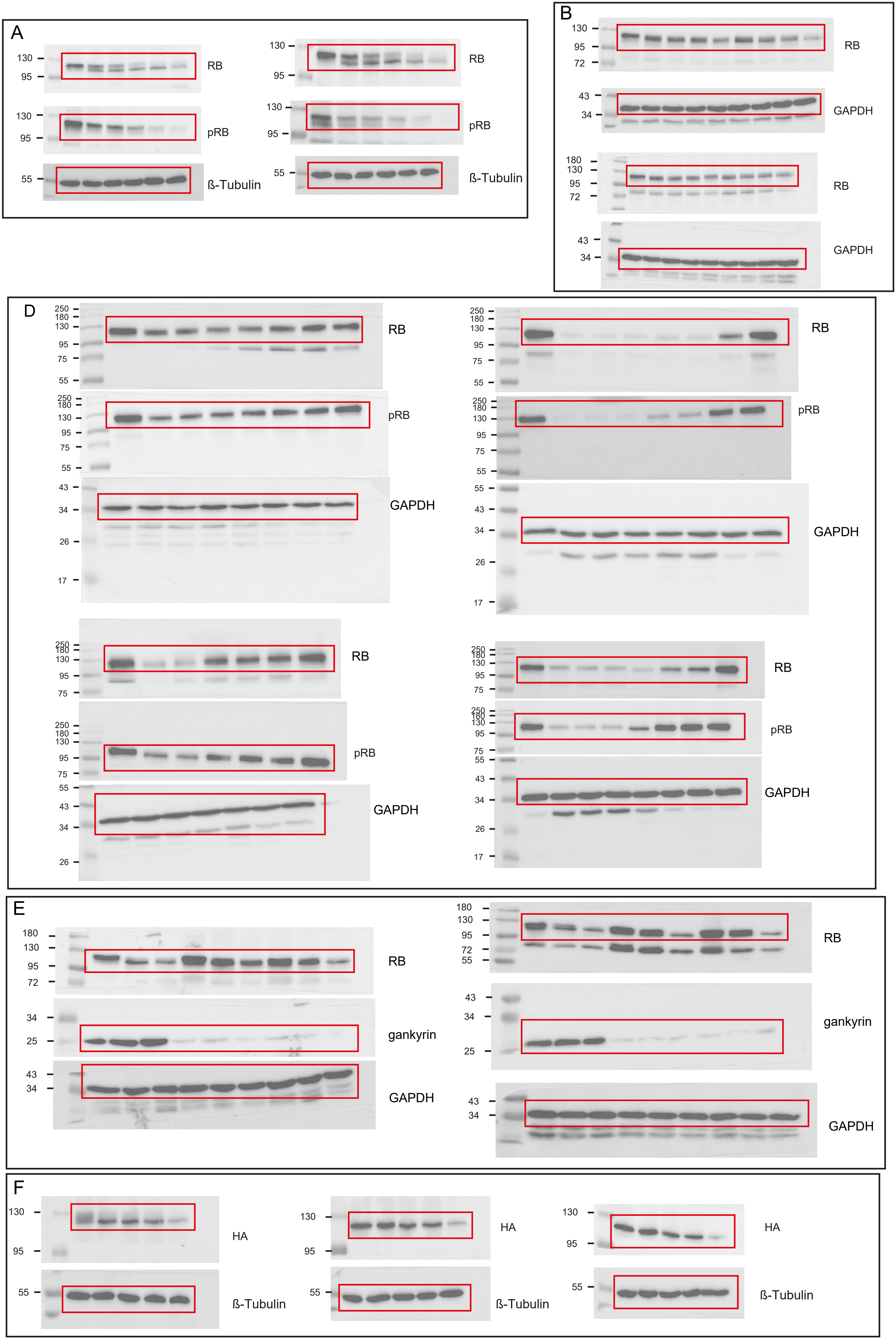

A
RB
pRB
ß-Tubulin
B
RB
GAPDH
D
RB
pRB
GAPDH
E
RB
gankyrin
GAPDH
F
HA
ß-Tubulin

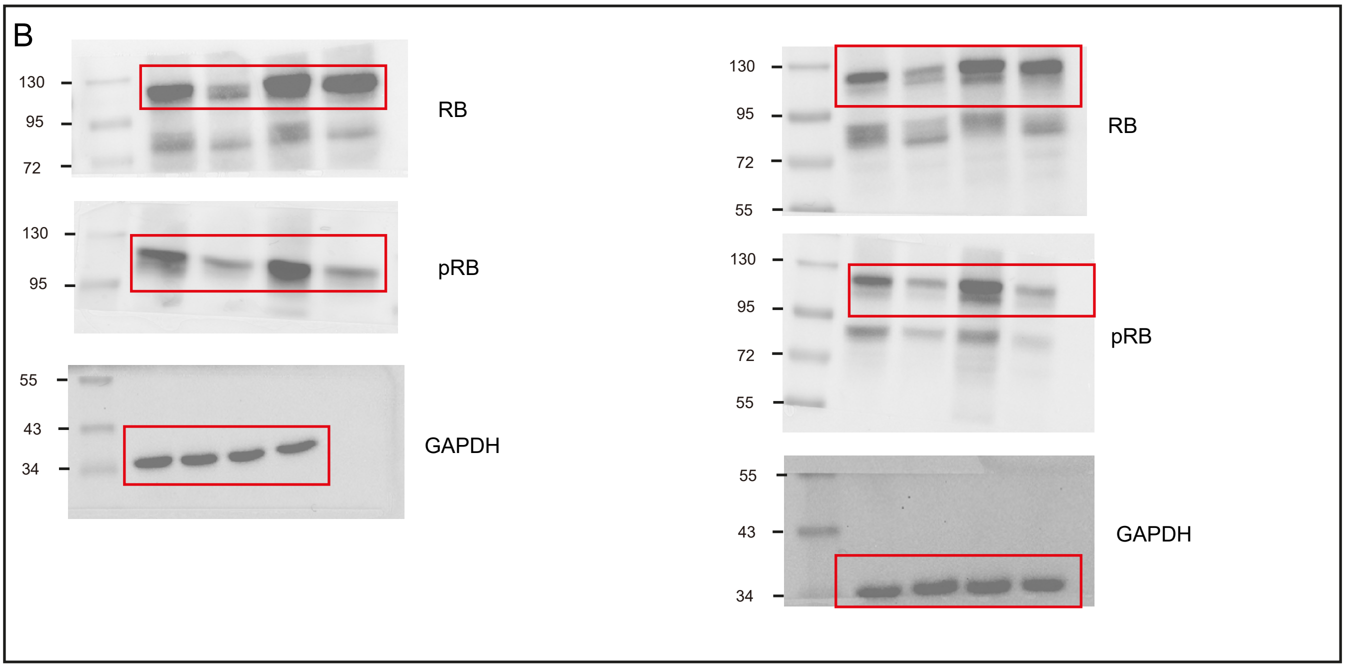
B
130
95
72
RB
130
95
pRB
55
43
34
GAPDH
130
95
72
55
RB
130
95
72
55
pRB
55
43
34
GAPDH

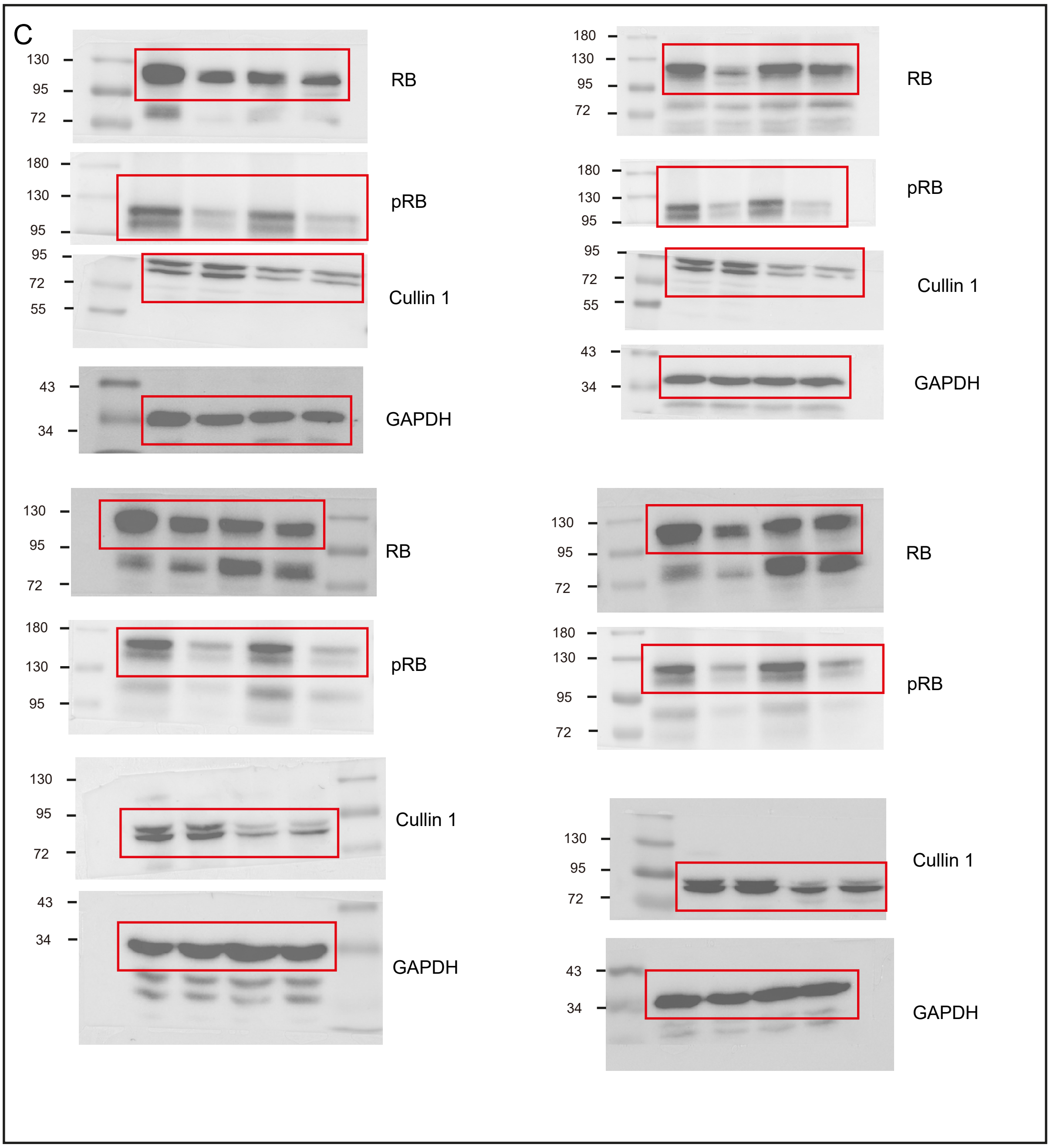
C
130
95
72
RB
180
130
95
pRB
95
72
55
Cullin 1
43
34
GAPDH
180
130
95
72
RB
180
130
95
pRB
95
72
55
Cullin 1
43
34
GAPDH
130
95
72
RB
180
130
95
pRB
130
95
72
Cullin 1
43
34
GAPDH
130
95
72
RB
180
130
95
72
pRB
130
95
72
Cullin 1
43
34
GAPDH

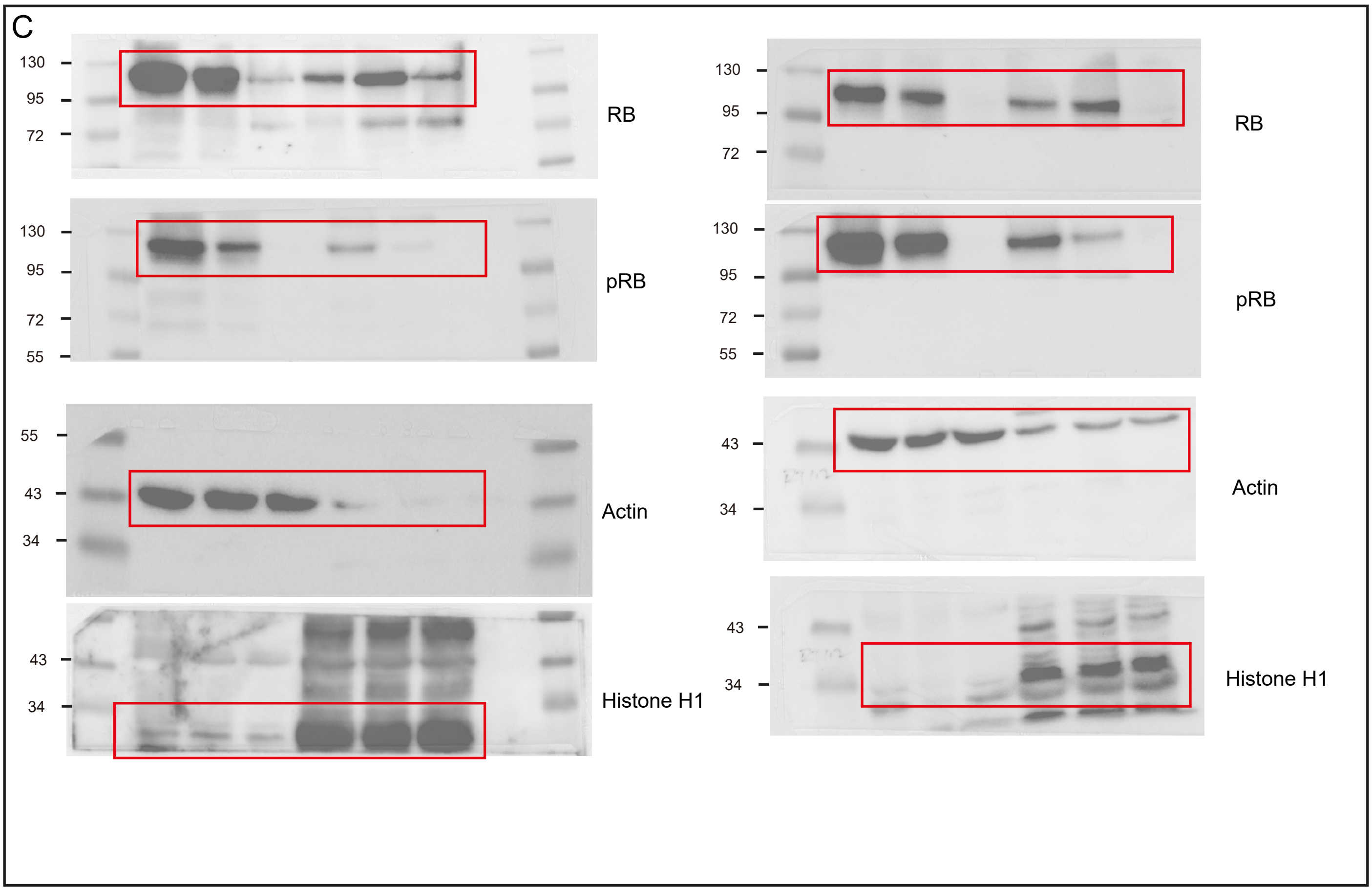
C
130
95
72
RB
130
95
72
55
pRB
55
43
34
Actin
43
34
Histone H1
130
95
72
RB
130
95
72
55
pRB
43
34
Actin
43
34
Histone H1

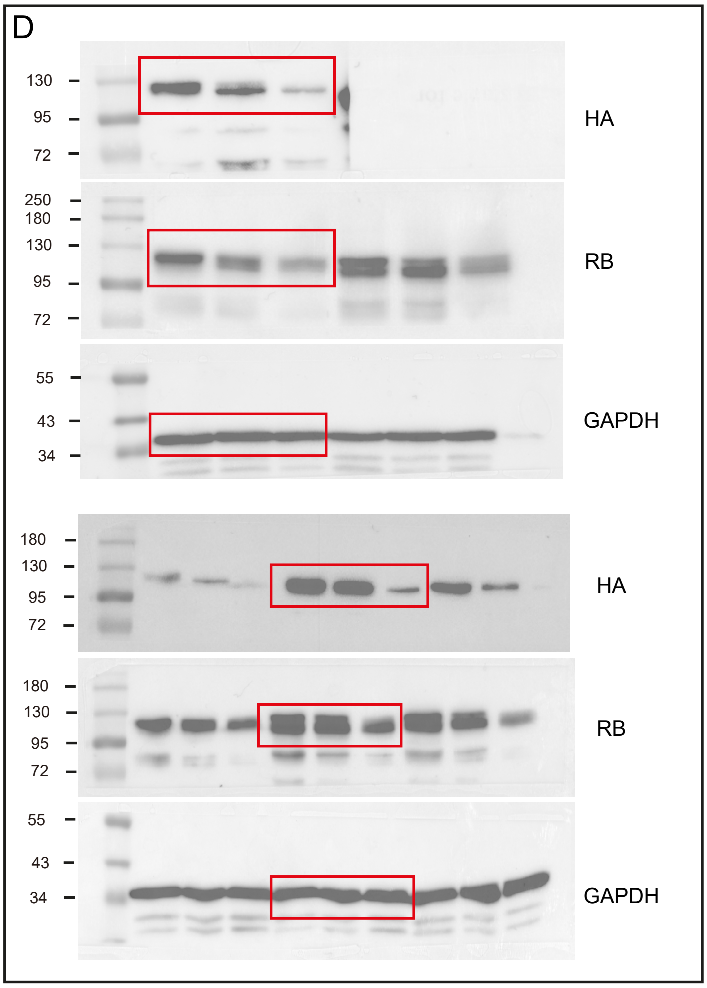
D
130
95
72
HA
250
180
130
95
72
RB
55
43
34
GAPDH
180
130
95
72
HA
180
130
95
72
RB
55
43
34
GAPDH

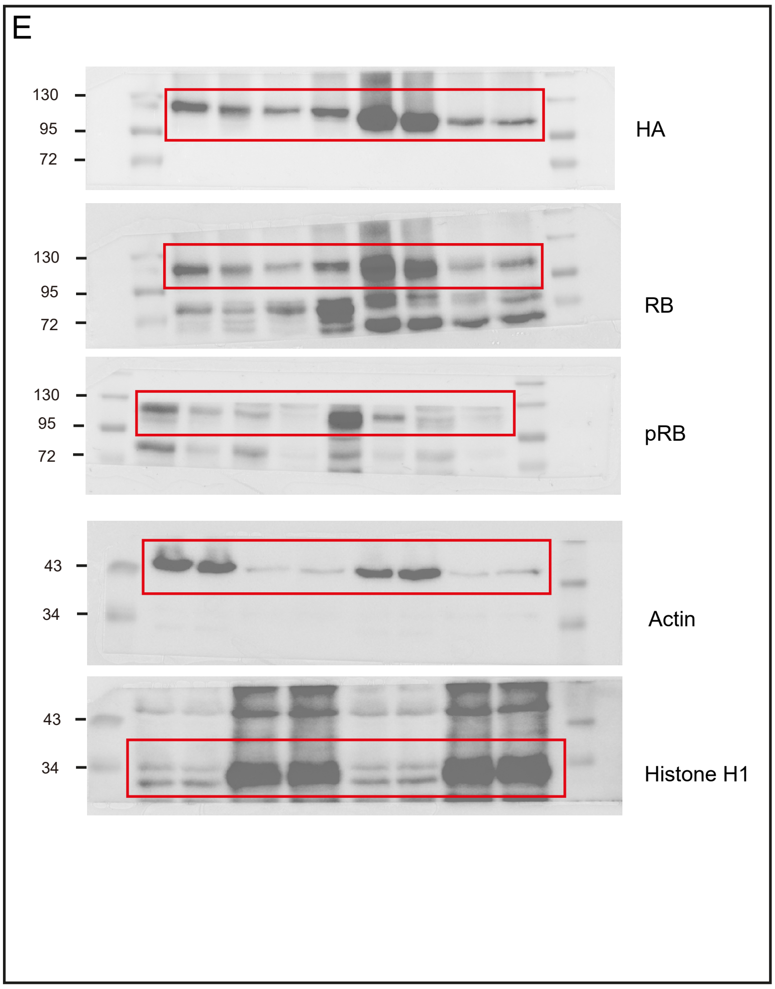
E
130
95
72
HA
130
95
72
RB
130
95
72
pRB
43
34
Actin
43
34
Histone H1

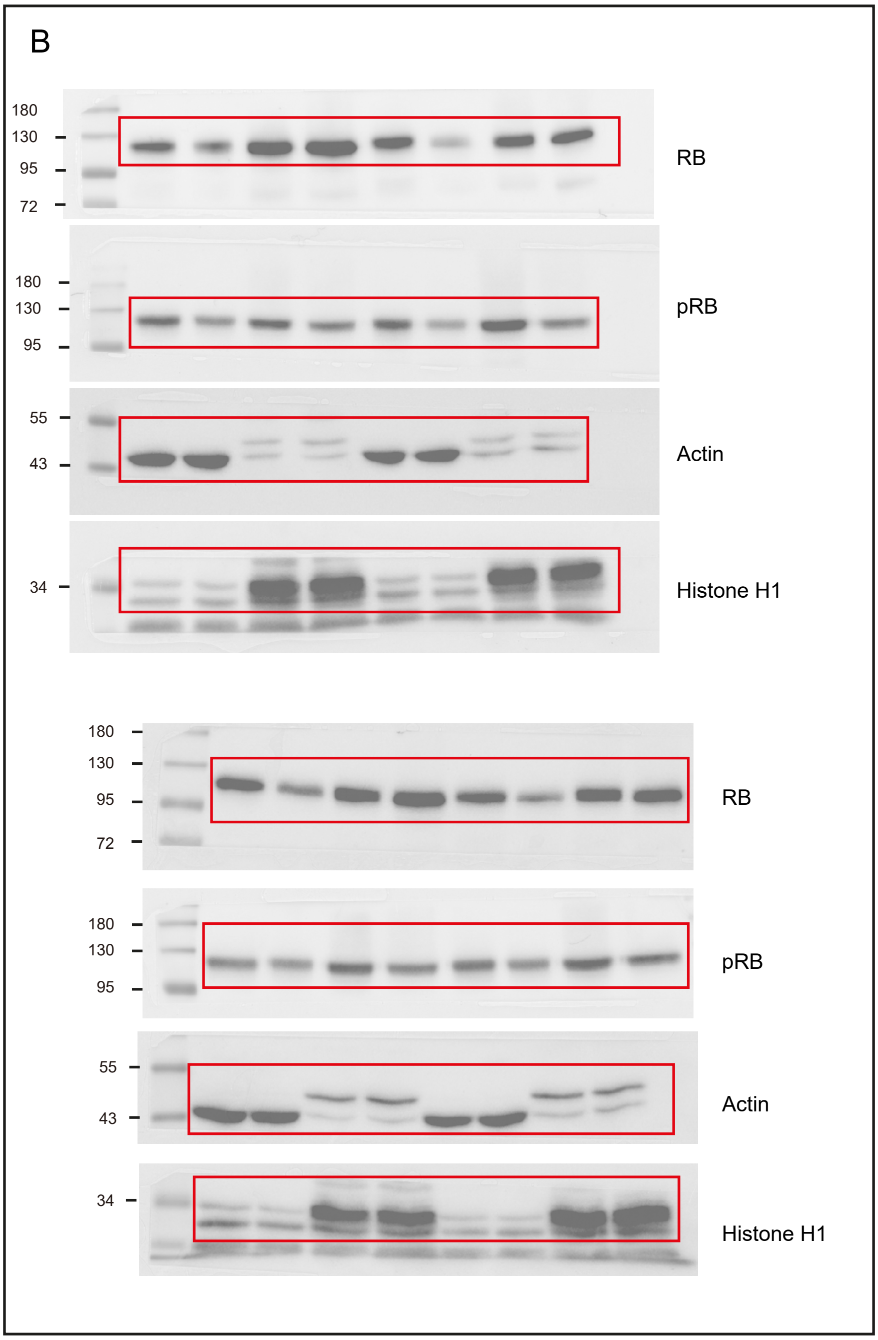
B
180
130
95
72
RB
180
130
95
pRB
55
43
Actin
34
Histone H1
180
130
95
72
RB
180
130
95
pRB
55
43
Actin
34
Histone H1

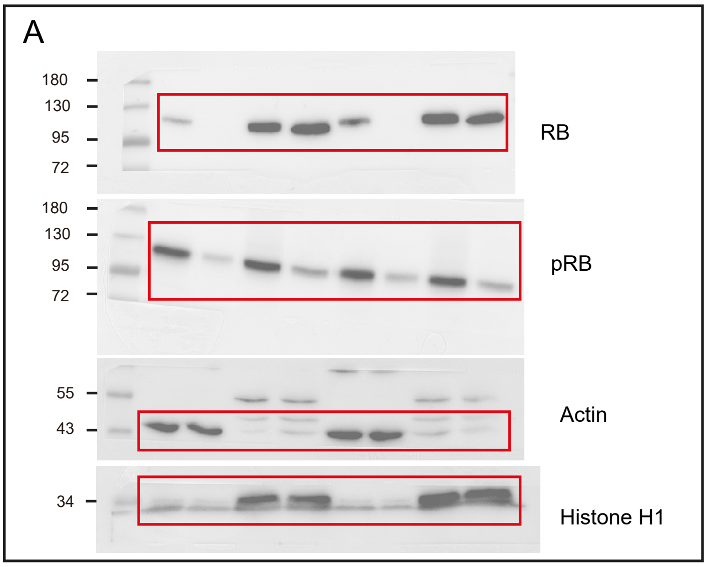
A
180
130
95
72
RB
180
130
95
72
pRB
55
43
Actin
34
Histone H1

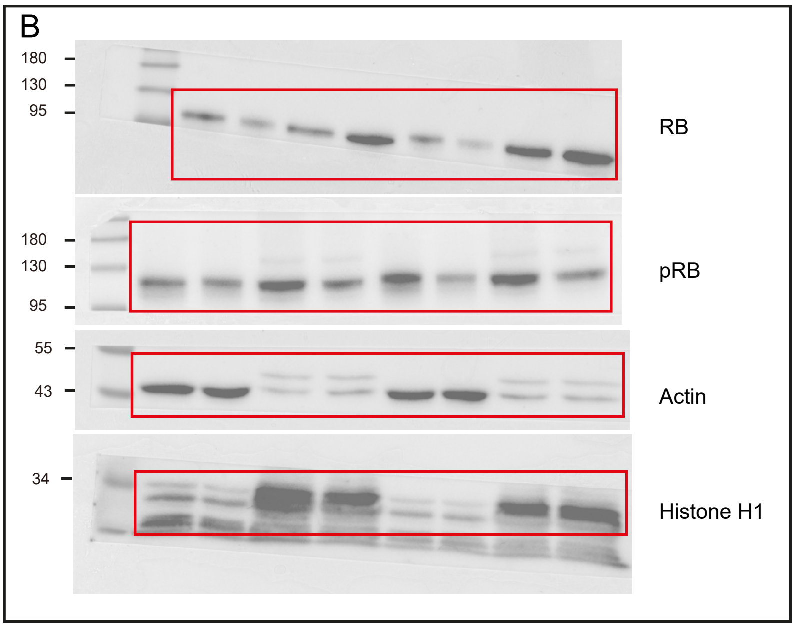
B
180
130
95
RB
180
130
95
pRB
55
43
Actin
34
Histone H1
